# SESN2 negatively regulates cell proliferation and casein synthesis by inhibition the amino acid-mediated mTORC1 pathway in cow mammary epithelial cells

**DOI:** 10.1038/s41598-018-22208-w

**Published:** 2018-03-02

**Authors:** Chaochao Luo, Shengguo Zhao, Muchen Zhang, Yanan Gao, Jiaqi Wang, Mark D. Hanigan, Nan Zheng

**Affiliations:** 1 State Key Laboratory of Animal Nutrition, Institute of Animal Sciences, Chinese Academy of Agricultural Sciences, Beijing, 100193 PR China; 2 Milk Risk Assessment Laboratory of Ministry of Agriculture, Institute of Animal Sciences, Chinese Academy of Agricultural Sciences, Beijing, 100193 PR China; 3 Department of Dairy Science, Virginia Tech, Blacksburg, VA 24061 USA

## Abstract

Amino acids (AA) are one of the key nutrients that regulate cell proliferation and casein synthesis in cow mammary epithelial cells (CMEC), but the mechanism of this regulation is not yet clear. In this study, the effect of SESN2 on AA-mediated cell proliferation and casein synthesis in CMEC was assessed. After 12 h of AA starvation, CMECs were cultured in the absence of all AA (AA−), in the presences of only essential AA (EAA+), or of all AA (AA+). Cell proliferation, casein expression, and activation of the mammalian target of rapamycin complex 1 (mTORC1) pathway were increased; but SESN2 expression was decreased in response to increased EAA or AA supply. Overexpressing or inhibiting SESN2 demonstrated that cell proliferation, casein expression, and activation of the mTORC1 pathway were all controlled by SESN2 expression. Furthermore, the increase in cell proliferation, casein expression, and activation of the mTORC1 pathway in response to AA supply was inhibited by overexpressing SESN2, and those effects were reversed by inhibiting SESN2. These results indicate that SESN2 is an important inhibitor of mTORC1 in CMEC blocking AA-mediated cell proliferation and casein synthesis.

## Introduction

Proliferation of cow mammary epithelial cells (CMEC) and casein synthesis by those cells are regulated by hormones (e.g. prolactin, insulin and glucocorticoids), nutrients (e.g. glucose and amino acids) and environmental stress (e.g. heat stress)^1–5^. Among the nutrients, amino acids (AA) are the most important as they are not only the building blocks of protein synthesis but also the regulators of cell proliferation and casein synthesis in mammalian epithelial cells^6,7^.

The main signaling pathway that mediates AA-induced cell proliferation and protein synthesis is the mammalian target of rapamycin complex 1 (mTORC1) pathway^8,9^. mTORC1 is the main regulatory factor in the pathway, and it is composed of mTOR, G protein β subunit-like protein (GβL), regulatory associated protein of mammalian target of rapamycin (Raptor), proline-rich Akt substrate of 40 kDa (PRAS40), and Deptor^10^. When AA are sufficient, mTORC1 is stimulated by an unknown signaling pathway and moves to the lysosomal surface from an undefined location, causing mTOR to be phosphorylated. Phosphorylated mTOR activates the downstream molecules, ribosomal protein S6 kinase 1 (S6K1) and eukaryotic translation initiation factor 4E binding protein 1 (4EBP1), which promotes participation in the translation process and protein synthesis^4,11,12^. The downstream actions of mTORC1 have been well characterized, but the mechanism of AA action on mTORC1 is poorly understood^13–15^.

Sestrins are a family of highly conserved, stress-inducible, metabolic regulators. In mammals, there are three members of the family: sestrin1 (SESN1), sestrin2 (SESN2) and sestrin3 (SESN3), of which, SESN2 is the most important^16–18^. Previous reports have shown that SESN2 can suppress reactive oxygen species arising from oxidative stress through its antioxidant function^19^. In addition to its antioxidant activity, SESN2 can activate adenosine monophosphate-activated protein kinase (AMPK), subsequently inhibiting the activation of mTORC1^20,21^. In human cells (mainly HELA and human embryonic kidney (HEK) 293 cells), SESN2 protein was found to respond to AA depletion (especially leucine) resulting in negative effects on the mTORC1 pathway. It has been reported that sestrins control mTORC1 signaling by inhibiting Rag GTPase^22–25^. Kimball *et al*. reported that sestrins/sestrin2 negatively regulated the amino-acid-sensing pathway upstream of mTORC1 by interacting with GTPase-activating proteins toward Rags 2 (GATOR2)^26^.

In this study, we investigated the function of SESN2 in the process of cell proliferation and casein synthesis associated with AA-mediated stimulation of the mTORC1 pathway in CMEC. We hypothesized that SESN2 mediated AA responses through inhibition of mTORC1 activation.

## Results

### SESN2 is negatively regulated by AA in CMECs

**Figure 1:**
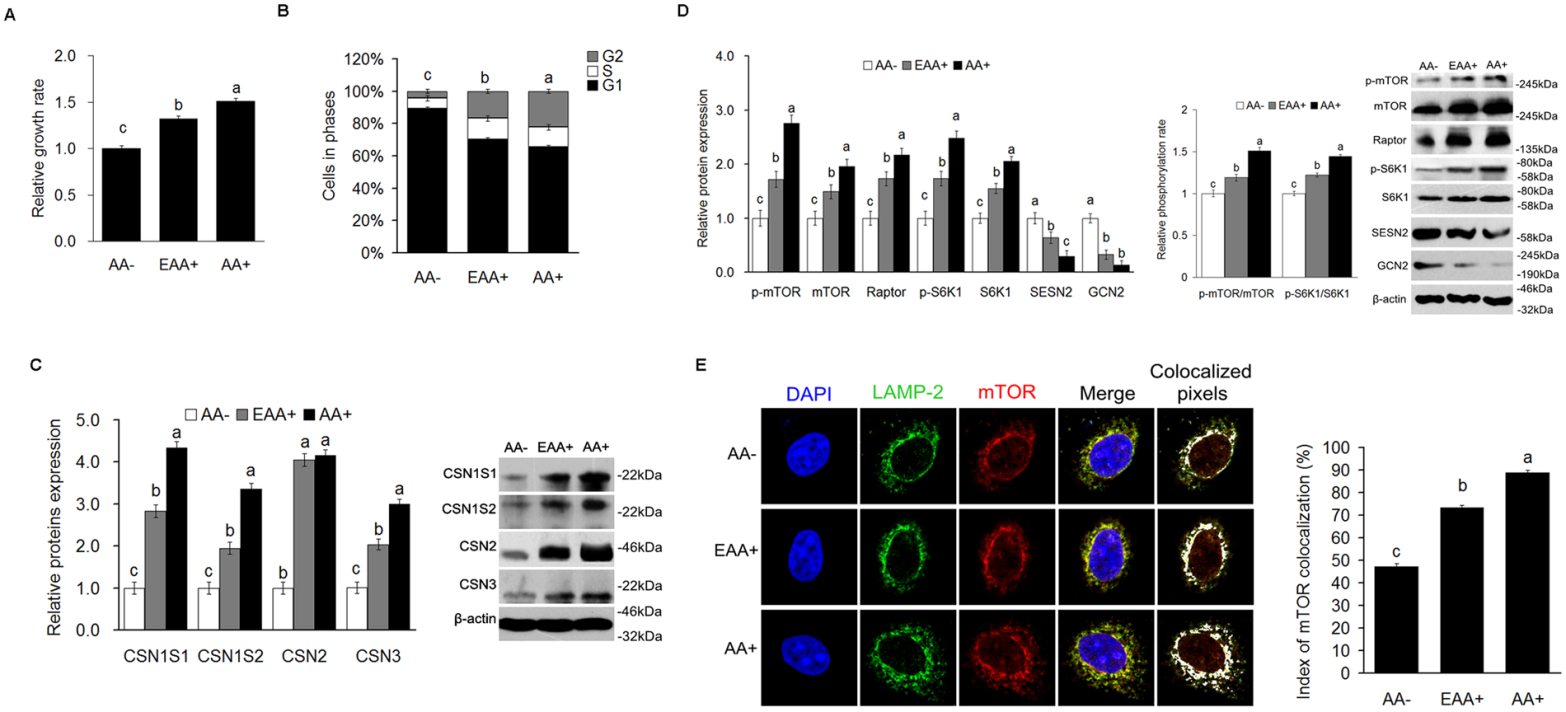
SESN2 is negatively regulated by AA in the process of AA-mediated regulation of cell proliferation and casein synthesis in CMEC. **(A)** Cell proliferation of CMEC treated with EAA or AA was analyzed by MTT assay, the data of “AA− group” was defined as “1”; **(B)** Cell cycle of CMEC treated with EAA or AA was analyzed by FC; **(C)** Expression of casein in CMEC treated with EAA or AA was analyzed by WB, the ratio value of “AA− group” was defined as “1”; **(D)** Expression of proteins related mTORC1 pathway, SESN2 and GCN2, and the phosphorylation ratio of mTOR and S6K1 in CMEC treated with EAA or AA was analyzed by WB, the ratio value of “AA− group” was defined as “1”; **(E)** Subcellular localization of mTOR in CMEC treated with EAA or AA for 6 h was analyzed by IF. Each bar represents the LSM ± SEM. In the bar charts, different superscript lowercase letters indicate significant difference (*p* < *0*.*05*). Uncropped images for all Western blots can be found in Supplementary Fig. 4.

To establish that the lactation cell model was regulated by AA, cell growth and cell cycle responses to AA were analyzed. The results showed that cell growth (Fig. 1A) and the proportion of cells in the G2+S stages (Fig. 1B) were increased (*P* < *0*.*05*) for essential AA (EAA+) and AA+ groups as compared to the AA- group. Treatment with EAA+ or AA+ also increased (*P* < *0*.*05*) alpha S1 casein (CSN1S1), alpha S2 casein (CSN1S2), beta casein (CSN2), kappa casein (CSN3), mTOR, p-mTOR, Raptor, S6K1 and p-S6K1 protein expression, and the ratio of p-S6K1 to S6K1, p-mTOR to mTOR (Fig. 1C,D), as well as lysosomal localization of mTOR (Fig. 1E and Supplementary Fig. 1). But the expression of SESN2 and general control nonderepressible 2 (GCN2) protein was decreased (*P* < *0*.*05*) when cells were treated with EAA+ or AA+ (Fig. 1D). The addition of non EAA (NEAA+) in the absence of EAA showed that cell growth, the expression of CSN2 and the activation of mTORC1 pathway (including expression of mTOR, S6K1, ratios of p-mTOR/mTOR and p-S6K1/S6K1, and lysosomal localization of mTOR) were increased (*P* < *0*.*05*) compared to the AA- group, but the responses were much smaller than for the EAA+ group (Supplementary Fig. 3A,B).

### SESN2 negatively regulates AA-mediated cell proliferation in CMEC

**Figure 2:**
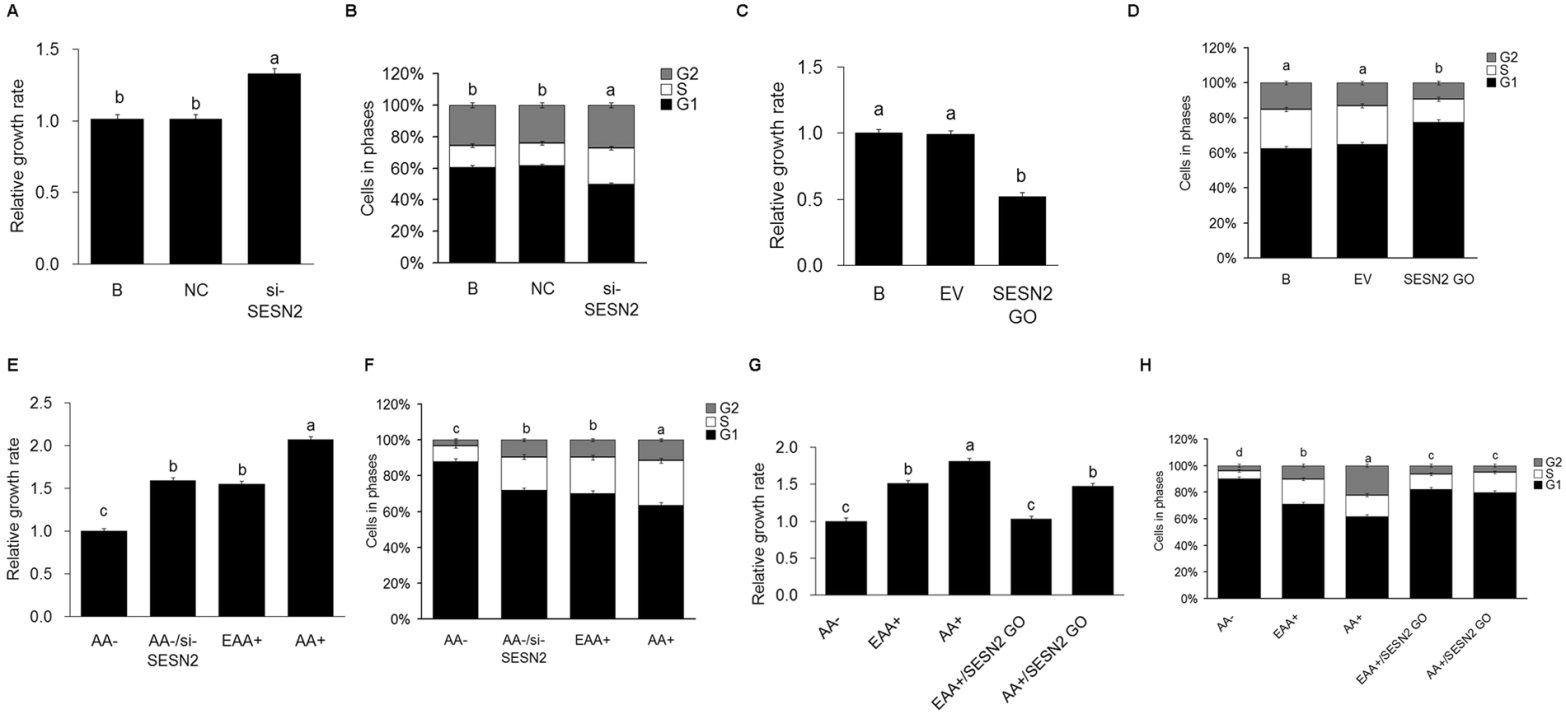
SESN2 negatively regulates AA-mediated cell proliferation in CMEC. **(A)** Cell proliferation of CMEC treated with si-SESN2 was analyzed by MTT assay, the date of the “B group” was defined as “1”; **(B)** Cell cycle of CMEC treated with si-SESN2 was analyzed by FC; **(C)** Cell proliferation of CMEC treated with SESN2 GO was analyzed by MTT assay, the date of the “B group” was defined as “1”; **(D)** Cell cycle of CMEC treated with SESN2 GO was analyzed by FC; **(E)** Cell proliferation of CMEC treated with AA−, AA−/si-SESN2, EAA+, and AA+ was analyzed by MTT assay, the date of “AA− group” was defined as “1”; **(F)** Cell cycle of CMEC treated with AA−, AA−/si-SESN2, EAA+, and AA+ was analyzed by FC; **(G)** Cell proliferation of CMEC treated with AA−, EAA+, AA+, EAA+/SESN2 GO, and AA+/SESN2 GO was analyzed by MTT assay, the date of “AA− group” was defined as “1”; **(H)** Cell cycle of CMEC treated with AA−, EAA+, AA+, EAA+/SESN2 GO, and AA+/SESN2 GO was analyzed by FC. Each bar represents the LSM±SEM. In the bar charts, different superscript lowercase letters indicate significant differences (*p* < *0*.*05*). B: Cells were no transfected; NC: Cells were transfected with negative control siRNA; EV: Cells were transfected with empty vector.

To investigate whether the expression of SESN2 mediates the effects of AA on cell proliferation, SESN2 was inhibited (si-SESN2) or overexpressed (SESN2 GO). The null transfection group (B) was used as blank control; the negative control siRNA transfected group (NC) was used as negative control; and the empty plasmid transfected group (EV) was used as empty plasmid control. Cell growth and cell cycle were analyzed. The results showed that cell growth (Fig. 2A) and the proportion of cells in G2 and S stages (Fig. 2B) were increased (*P* < *0*.*05*) when SESN2 expression was inhibited, and the reverse when it was overexpressed (Fig. 2C,D).

To investigate whether AA regulate cell growth and the cell cycle via SESN2, CMEC were treated with AA−, EAA+, AA+, AA−/si-SESN2, EAA+/SESN2 GO and AA+/SESN2 GO; and cell growth and cell cycle were analyzed. The results showed that decreases in cell growth and decreases in the proportion of cells in the G2 and S stages caused by AA starvation were blocked (*P* < *0*.*05*) when SESN2 was inhibited (Fig. 2E,F) resulting in growth and cell cycle patterns equivalent to those of the EAA+ treatment. Conversely, the stimulatory effects of AA+ on cell growth and proportions of cells in the G2+S phases were inhibited (*P* < *0*.*05*) by SESN2 overexpression (Fig. 2G,H).

### SESN2 negatively regulates AA-mediated casein synthesis in CMEC

**Figure 3:**
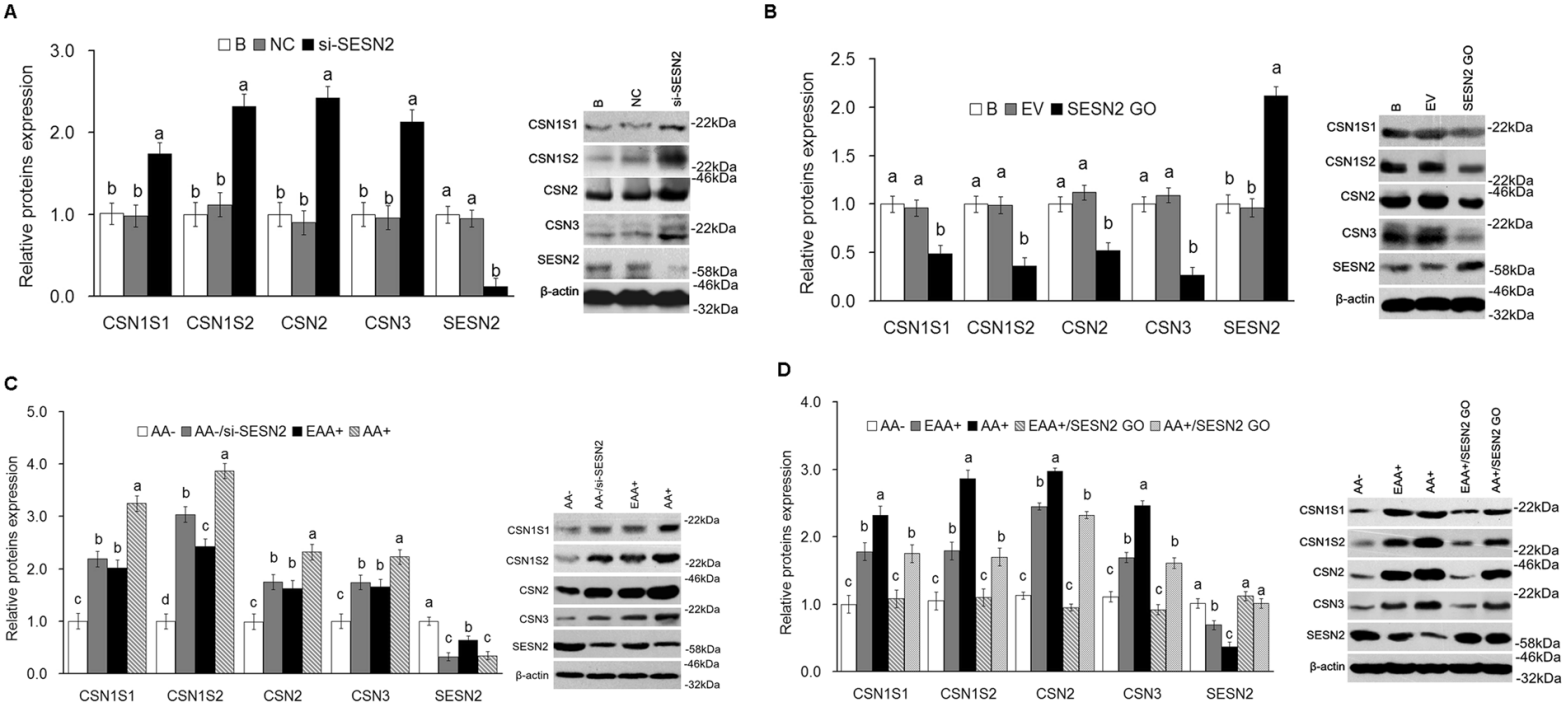
SESN2 negatively regulates AA-mediated casein synthesis in CMEC. **(A)** Expression of casein in CMEC treated with si-SESN2 was analyzed by WB, the ratio value of “B group” was defined as “1”; **(B)** Expression of casein in CMEC treated with SESN2 GO was analyzed by WB, the ratio value of “B group” was defined as “1”; **(C)** Expression of casein in CMEC treated with AA−, AA−/si-SESN2, EAA+, and AA+ was analyzed by WB, the ratio value of “AA− group” was defined as “1”; **(D)** Expression of casein in CMEC treated with AA−, EAA+, AA+, EAA+/SESN2 GO, and AA+/SESN2 GO was analyzed by WB, the ratio value of “AA− group” was defined as “1”. Each bar represents the LSM ± SEM. In the bar charts, different superscript lowercase letters indicate significant differences (*p* < *0*.*05*). B: Cells were no transfected; NC: Cells were transfected with negative control siRNA; EV: Cells were transfected with empty vector. Uncropped images for all Western blots can be found in Supplementary Fig. 4.

The effect of SESN2 on casein synthesis was analyzed. The results showed that expression of CSN1S1, CSN1S2, CSN2 and CSN3 was increased (*P* < *0*.*05*) (Fig. 3A) in cells with SESN2 inhibited and decreased (*P* < *0*.*05*) (Fig. 3B) in cells overexpressing SESN2.

AA starvation decreased expression of CSN1S1, CSN1S2, CSN2 and CSN3; but this decrease was rescued (*P* < *0*.*05*) by inhibition of SESN2 (Fig. 3C), and AA stimulating effects were inhibited (*P* < *0*.*05*) by SESN2 overexpression (Fig. 3D).

### SESN2 negatively regulates activation of AA-mediated mTORC1 pathway in CMEC

**Figure 4:**
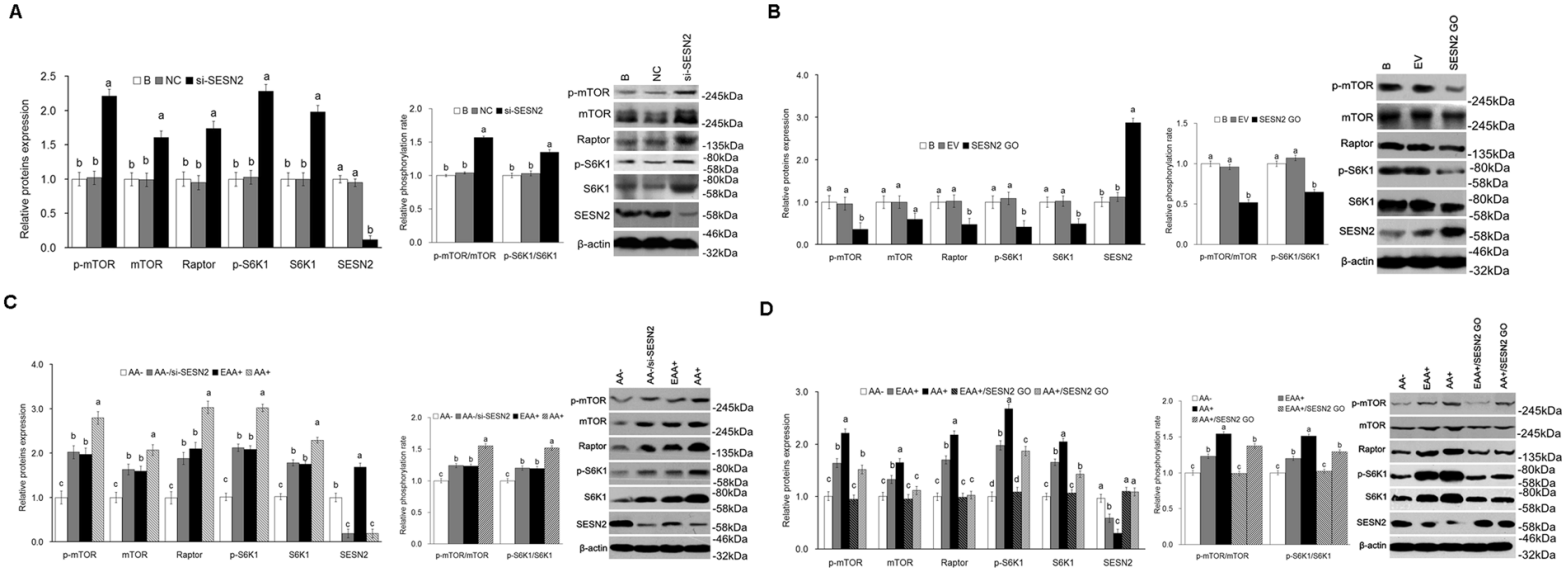
SESN2 negatively regulates AA-mediated mTORC1 activation in CMEC. **(A)** Expression of proteins related mTORC1 pathway and the phosphorylation ratio of mTOR and S6K1 in CMEC treated with si-SESN2 was analyzed by WB, the ratio value of “B group” was defined as “1”; **(B**) Expression of proteins related mTORC1 pathway and the phosphorylation ratio of mTOR and S6K1 in CMEC treated with SESN2 GO was analyzed by WB, the ratio value of “B group” was defined as “1”; **(C)** Expression of proteins related mTORC1 pathway and the phosphorylation ratio of mTOR and S6K1 in CMEC treated with AA−, AA−/si-SESN2, EAA+, and AA+ was analyzed by WB, the ratio value of “AA− group” was defined as “1”; **(D)** Expression of proteins related mTORC1 pathway and the phosphorylation ratio of mTOR and S6K1 in CMEC treated with AA−, EAA+, AA+, EAA+/SESN2 GO, and AA+/SESN2 GO was analyzed by WB, the ratio value of “AA− group” was defined as “1”. Each bar represents the LSM ± SEM. In the bar charts, different superscript lowercase letters indicate significant differences (*p* < *0*.*05*). B: Cells were no transfected; NC: Cells were transfected with negative control siRNA; EV: Cells were transfected with empty vector. Uncropped images for all Western blots can be found in Supplementary Fig. 4.

Expression of mTOR, p-mTOR, Raptor, S6K1 and p-S6K1, as well as the ratio of p-S6K1 to S6K1, p-mTOR to mTOR was increased (*P* < *0*.*05*) (Fig. 4A) in cells with SESN2 inhibited and decreased (*P* < *0*.*05*) (Fig. 4B) in cells overexpressing SESN2. Additionally, the decrease in expression of mTOR, p-mTOR, Raptor, S6K1 and p-S6K1, as well as the ratio of p-S6K1 to S6K1, p-mTOR to mTOR caused by AA starvation was rescued (*P* < *0*.*05*) by SESN2 inhibition (Fig. 4C). AA stimulation increased (*P* < *0*.*05*) expression of p-mTOR, Raptor, S6K1 and p-S6K1, as well as the ratio of p-S6K1 to S6K1, p-mTOR to mTOR; and the increase was inhibited (*P* < *0*.*05*) by SESN2 overexpression (Fig. 4D). The independent additions of leucine (Leu), isoleucine (Ile), valine (Val), methionine (Met) or glutamine (Gln) to the AA- media down regulated (*P* < *0*.*05*) the expression of SESN2 and up regulated (*P* < *0*.*05*) the mTORC1 signaling pathway (Supplementary Fig. 2), however, the effects were not as great as AA + indicating that some additivity of responses must exist. The independent additions of arginine (Arg) or lysine (Lys) to AA− media did not affect (*P* > *0*.*05*) expression of SESN2 but up regulated (*P* < *0*.*05*) the mTORC1 signaling pathway (Supplementary Fig. 3B); and the independent additions of proline (Pro) or Alanine (Ala) to the AA− media did not affect (*P* > *0*.*05*) the expression of SESN2 or the mTORC1 signaling pathway (Supplementary Fig. 3B).

**Figure 5:**
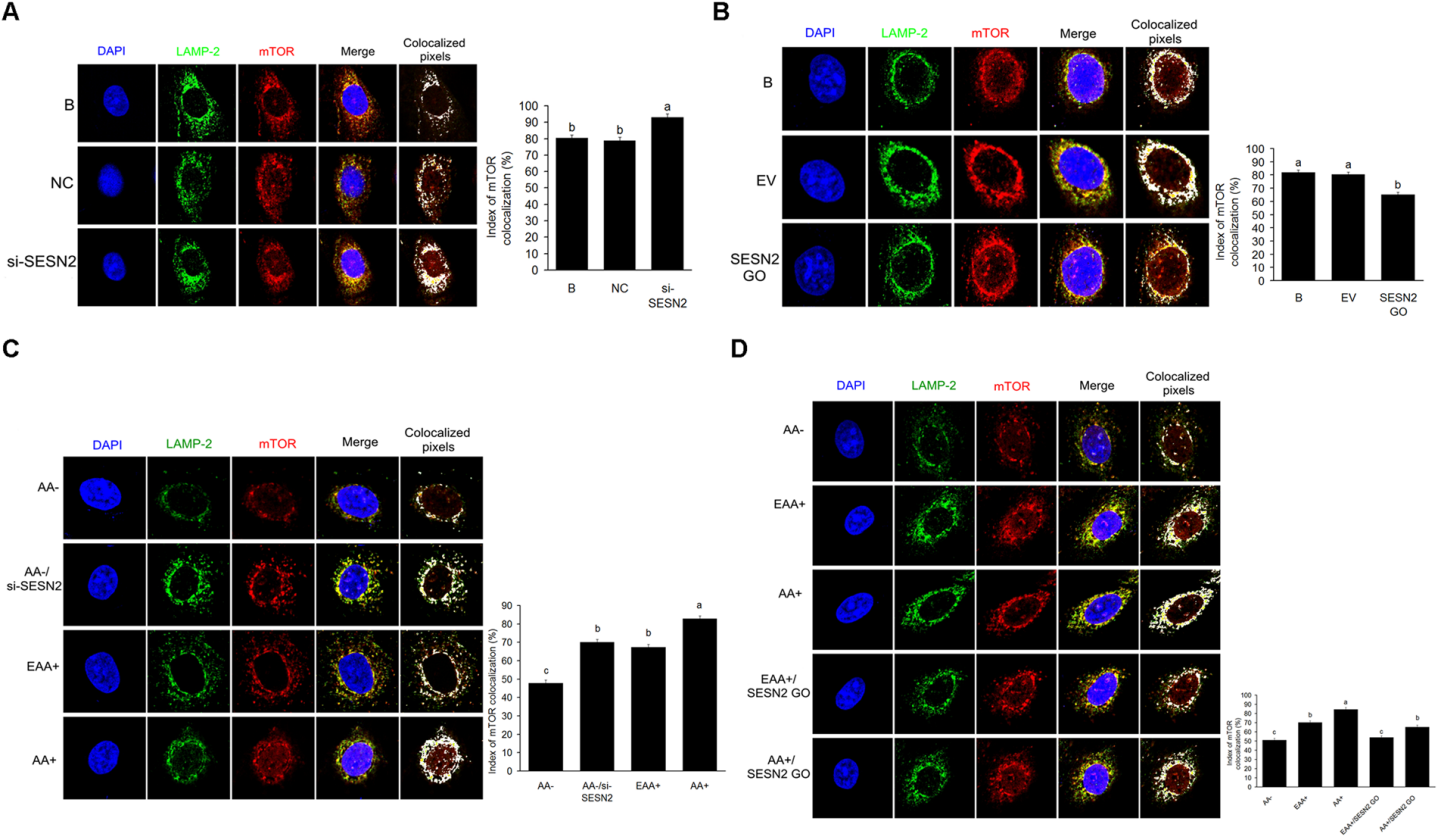
SESN2 negatively regulates AA-mediated lysosomal localization of mTOR in CMEC. **(A)** Subcellular localization of mTOR in CMEC treated with si-SESN2 was analyzed by IF; **(B)** Subcellular localization of mTOR in CMEC treated with SESN2 GO was analyzed by IF; **(C)** Subcellular localization of mTOR in CMEC treated with AA−, AA−/si-SESN2, EAA+, and AA+ was analyzed by IF; **(D)** Subcellular localization of mTOR in CMEC treated with AA−, EAA+, AA+, EAA+/SESN2 GO, and AA+/SESN2 GO was analyzed by IF. Each bar represents the LSM ± SEM. In the bar charts, different superscript lowercase letters indicate significant differences (*p* < *0*.*05*). B: Cells were no transfected; NC: Cells were transfected with negative control siRNA; EV: Cells were transfected with empty vector.

Lysosomal localization of mTOR was increased (*P* < *0*.*05*) (Fig. 5A) in cells with SESN2 inhibited, and decreased (*P* < *0*.*05*) (Fig. 5B) in cells overexpressing SESN2. Additionally, lysosomal localization of mTOR was decreased (*P* < *0*.*05*) by AA starvation, and this decrease was rescued (*P* < *0*.*05*) by SESN2 inhibition (Fig. 5C). Lysosomal localization of mTOR in response to increased AA was inhibited (*P* < *0*.*05*) by SESN2 overexpression (Fig. 5D).

### SESN2 negatively regulates cell proliferation and casein synthesis via mTORC1 pathway in CMEC

**Figure 6:**
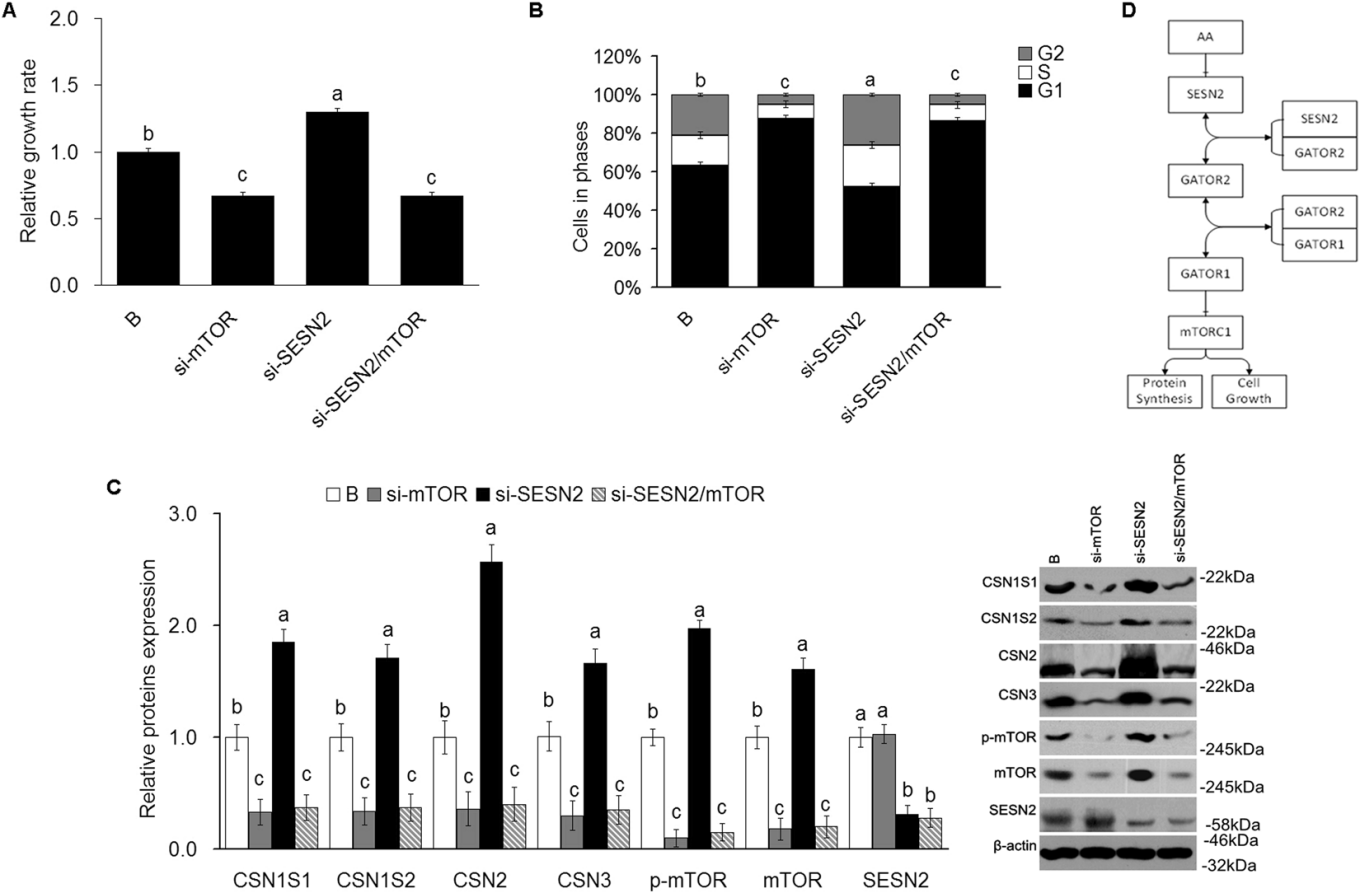
SESN2 negatively regulates cell proliferation and casein synthesis via mTORC1 pathway in CMEC. **(A)** Cell proliferation of CMEC treated with si-SESN2, si-mTOR, and si-SESN2/si-mTOR was analyzed by MTT assay, the date of “B group” was defined as “1”; **(B)** Cell cycle of CMEC treated with si-SESN2, si-mTOR, and si-SESN2/si-mTOR was analyzed by FC; **(C)** Expression of casein in CMEC treated with si-SESN2, si-mTOR, and si-SESN2/si-mTOR was analyzed by WB, the ratio value of “B group” was defined as “1”. **(D)** The model of SESN2 negative regulates the AA-mediated cell proliferation and casein synthesis via inhibiting mTORC1 pathway. Each bar represents the LSM ± SEM. In the bar charts, different superscript lowercase letters indicate significant differences (*p* < *0*.*05*). B: Cells were no transfected. Uncropped images for all Western blots can be found in Supplementary Fig. 4.

Cell growth was decreased (*P* < *0*.*05*) and the proportion of cells in the G2 and S stages decreased (*P* < *0*.*05*) when mTOR gene expression was inhibited. mTOR inhibition also prevented (*P* < *0*.*05*) cell growth and G2 and S stage changes caused by SESN2 inhibition (Fig. 6A,B). Inhibition of mTOR mRNA expression inhibited (*P* < *0*.*05*) expression of CSN1S1, CSN1S2, CSN2 and CSN3, and inhibition of SESN2 increased each; however, inhibition of mTOR blocked (*P* < *0*.*05*) the SESN2 responses (Fig. 6C).

## Discussion

AA regulate cell proliferation and milk protein synthesis primarily through their regulation of the mTORC1 pathway and subsequent effects on translation^27–29^. The observed increases in mTORC1 phosphorylation, cell growth, and casein synthesis in response to AA supplementation (Fig. 1 and Supplementary Fig. 1) herein were all consistent with previous research^2,30,31^ despite the lack of inclusion of lactogenic hormones in the media. Although Osorio *et al*.^1^ indicated that lactogenic hormones are required for regulation of casein expression, there is some dichotomy between requirements to initiate lactation from a nonlactating state and maintenance of an existing lactational state, particularly in ruminants. Clearly ruminants are much less sensitive to prolactin than rodents, and the role of prolactin may be more associated with long-term maintenance of the cell numbers^32^. Ruminants also appear to have little to no sensitivity to cortisol after lactation has been initiated^33^. In our study, the CMEC were isolated and cultured from the udder of a mid-lactation healthy Holstein dairy cow, i.e. expression of caseins had been initiated. Additionally, CMEC were cultured prior to experiments in DMEM/F12 media containing 10% FBS. As FBS contains insulin, cortisol, and prolactin^34^, maintenance of expression of caseins by the cells would have been supported by the FBS during cell growth^35–37^. The FBS was removed during the experiments with no hormone replacement to avoid the potential of saturating the mechanisms utilized by AA through the inclusion of hormones at supraphysiological concentrations. While one cannot rule out any effects of hormone removal, the CMEC continued to transcribe and translate caseins for the 18 h period required to complete the casein measurements. As identical hormone conditions were used for all treatments, any potential effects of hormone removal would not have affected treatment comparisons. Additional work will be required to assess interactions among these hormones and AA.

The activation of mTORC1 is important for cell growth and protein synthesis, but how AA affect mTORC1 is still uncertain. A number of independent reports have identified several potential pieces of the mechanism, but a clear picture has not yet emerged. They include AA activation of mTORC1 via protein phosphatase 2 oligomer containing the PR61ε-targeting subunit (PP2AT61ε), *Ste20* family kinase MAP4K3^38^; an inside-out mechanism^39^; a G protein coupled receptor (GPCR) T1R1/T1R3^40^; PB1-containing kinase MEEK3/p38δ/p62/E3-ubiquitin ligase TRAF6^41^; and Sestrins/GATOR2/GATOR1^22,25^. We have shown herein that in CMEC, the expression of SESN2 was significantly decreased in response to EAA or AA supply (Fig. 1D), which is consistent with the results of Chantranupong *et al*.^22^ and Parmigiani *et al*. ^23^ in HEK 293 T and Kimball *et al*.^26^ in mouse embryonic fibroblasts (MEF) cells. These results support SESN2 as the mechanism of regulation of AA effects on the mTORC1 pathway in CMEC.

SESN2, the product of hypoxia-inducible gene 95 (Hi95), is a member of the sestrins family^42^. Previous studies have shown that the expression of SESN2 was induced by DNA damage, oxidative stress and glucose starvation^43,44^. Expression of SESN2 was stimulated by genotoxic stress via tumor suppressor p53^42,45^ in HEK293 and MCF-7 cells; by glucose starvation through activation of transcription factor 4 (ATF4) and nuclear factor (erythroid-derived 2)-like 2 (NRF2) transcription factors^43,46^ in human lung adenocarcinoma H1299 and H460 cell lines and MEF; and by AA deprivation acting through GCN2, ATF4 in MEF cells^47^. In this study, we showed that the expression of SESN2 and GCN2 was significantly decreased in response to increased EAA or AA supply (Fig. 1D), suggesting that AA starvation stimulates SESN2 and GCN2 expression. The AA response was elicited by individual AA including Leu, Ile, Val, Met and Gln (Supplementary Fig. 2), which is consistent with the findings of Ye *et al*. in MEF^44^.

Sestrins (in particular, SESN2) play an important metabolic regulation role in mammals. Early studies concluded that SESN2 inhibited the mTORC1 pathway through AMPK-mTORC1 signaling^48,49^. However, recent studies demonstrated that SESN2 regulates mTORC1 in mouse embryonic fibroblast (MEF), HEK-293T, and Hela cells^22,26,50^ by binding to GATOR2, which disrupts the GATOR1/GATOR2 complex thus releasing GATOR1 to inhibit mTORC1^24,25^. Kimball *et al*. reported that SESN2 regulates the mTORC1 pathway depending on its phosphorylation state which is driven by Leu deprivation, and no other EAA^26^. Control can also be exerted through changes in SESN2 gene expression. Ye *et al*. and Wolfson *et al*. showed that Ile, lysine, Val, Gln and Met regulate the expression of SESN2 which, in turn, inhibits the mTORC1 pathway^25,47^. In this study, we found that Leu, Ile, Val, Met and Gln independently regulated the mTORC1 pathway through changes in expression of SESN2 (Fig. 1D and Supplementary Fig. 2), but Arg and Lys regulation of the mTORC1 pathway was not through changes in expression of SESN2 (Supplementary Fig. 3B). These findings are consistent with the results of Ye *et al*.^47^, and collectively indicate SESN2 can respond to many, but not all, individual AA in CMEC.

The current work cannot differentiate between the above alternatively proposed upstream mechanisms; however, it clearly demonstrates that SESN2 is involved in mediating AA effects on mTORC1 and that SESN2 lies upstream of mTORC1. It also extends prior observations in other species and cell types to CMEC. Cell proliferation, casein synthesis and activation of the mTORC1 pathway were all significantly increased with SENS2 inhibition and significantly decreased with SESN2 overexpression (Figs 2A,B, 3A,B, 4A,B and 5B). Additionally, responses to AA (EAA+ and AA+) were significantly blocked when SESN2 was overexpressed (Figs 2D, 3C,D, 4A,B and 5C,D). Finally, the effects of SESN2 over or under expression were significantly blocked when mTORC1 protein expression was inhibited (Fig. 6). The potential system is schematically summarized in Fig. 6D.

Because AA act as both a substrate and a regulator of protein synthesis, it is of interest to discern the effects of each mechanism. From Figs 3 and 4, it is clear that essentially all of the mTORC1 and casein responses to EAA (as compared to AA−) and total AA (as compared to EAA+) were significantly blocked by overexpression of SESN2, supporting the conclusion that cell signaling is the primary controller of rates of protein synthesis. Had AA exerted any control as a substrate, one would expect to see a response to the addition of AA independent of the expression levels of SESN2, which was not the case.

The results also demonstrate that NEAA are impacting SESN2 with subsequent effects on mTORC1 signaling, cellular growth rates, and casein synthesis. Appuhamy *et al*. and Arriola *et al*. observed independent responses to multiple EAA^6,51^; however, preliminary screenings of NEAA, using a bovine tissue slice model, did not show any responses to the NEAA^52,53^. The current results provide evidence that the NEAA should be screened for regulatory activity and possibly included in models of AA effects on mammary function^54^. This is an important finding as it potentially adds considerable complexity to the system where the response surface may have as many as 20 dimensions, thus making parameterization of the system exceedingly difficult.

In summary, the present work shows that SESN2 is an important negative regulatory factor which was induced by EAA and NEAA starvation, and it negatively regulates AA-mediated cell proliferation and casein synthesis via inhibition of the mTORC1 pathway in CMEC.

## Materials and Methods

All experiments involving dairy cattle were conducted according to the principles of the Chinese Academy of Agricultural Sciences Animal Care and Use Committee (Beijing, China), which approved study protocols.

### Cell preparation and treatments

Mammary gland tissue was dissected from the udder of mid-lactation healthy Holstein dairy cow and cut into 1 mm^3^ pieces using sterile techniques. The explants were plated into 25 cm^2^ flasks (Corning, USA) coated with 5 mg/ml sterile rat tail collagen type II (diluted with 0.006 M acetic acid) (Shengyou, Hangzhou, China) and cultured with DMEM/F12 media (11320033, Gibco, USA) containing 10% fetal bovine serum (FBS), 10,000U of penicillin, 10,000U of streptomycin, and 25 mg/mL of amphotericin B in an incubator at 37 °C in and atmosphere of 5% CO_2_. At 80% confluence, the explants were removed from the culture. Epithelial and fibroblast cells were segregated using 0.25% trypsin and 0.15% trypsin plus 0.02% ethylenediaminetetraacetic acid (EDTA) (C0203, Beyotime, China) as previously described^55^ yielding primary cow mammary epithelial cells (CMEC).

For the experiments, CMEC were plated into 6 well plates at a density of 1.0 × 10^5^ cells per well and cultured with DMEM/12 containing 10% FBS. When they reached 80% confluence, cells were starved of AA (cells cultured with DMEM/F12 media without FBS and devoid of all AA) for 12 h, and then treated with DMEM/F12 media without FBS and devoid of all AA (AA−), containing only essential AA (EAA, containing lysine, methionine, valine, leucine, isoleucine, tryptophan, phenylalanine and threonine in this manuscript) at normal DMEM/F12 concentrations (EAA+), or complete DMEM/F12 (AA+) for 6 h. For cell growth tests, treatment duration was 24 h. The concentrations of each EAA in the EAA+ treatment were those of normal DMEM/F12 medium. All AA used in the study were obtained from Sigma (San Francisco, USA). The main effects of AA were also assessed with overexpression (SESN2 GO) or inhibition of SESN2 (si-SESN2) yielding the additional treatments of AA−/si-SESN2, EAA+/SESN2 GO, AA+/SESN2GO, si-mTOR and si-SESN2/si-mTOR.

### Cell growth

Cell growth was analyzed with 3-(4,5)-dimethylthiahiazo (-z-y1)-3, 5-di-phenytetrazoliumromide (MTT) (M5655, Sigma, San Francisco, USA). CMEC were plated in 96 well plates at a density of 5.0 × 10^4^ cells/ml and cultured with DMEM/12 containing 10% FBS. After 12 h, media were switched to an AA and FBS free form followed by treatment media (DMEM/F12 media devoid of all AA, containing only EAA or complete DMEM/F12, Leagene Biotechnology, Beijing, China) for 24 h. At the end of the treatment period, MTT (5 mg/mL diluted in PBS) was added to the media at a final concentration of 0. 5 mg/mL and the cells were cultured for an additional 4 h. The media were then discarded, and 150 μl of DMSO (D2650, Sigma, USA) was added to each well. The plates were shaken for 10 minutes at room temperature, and the absorbance at 450 nm was determined using an automated enzyme-linked immunosorbent assay (ELISA) reader (MD, Spectramax M3, USA)^56^. The relative growth rate of the treated cells as compared to the control cells (AA− group) was reported. This experiment was repeated with three different batches of cells.

### Cell cycle

The cell cycle was analyzed by flow cytometry (FC) as previously reported^57^. The CMEC were plated into 6 well plates and treated as described for cell growth analysis, but the density of plated cells was 1.0 × 10^5^ cells per well. Cells were digested with 0.25% trypsin-0.02% EDTA, harvested, washed 3 times with PBS (135 mM NaCl, 4.7 mM KCl, 10 mM Na_2_HPO_4_, 2 mM NaH_2_PO_4_, pH = 7.4), fixed overnight with ice-cold 70% ethanol at 4 °C, and washed 3 times with PBS followed by incubation with 1 mL RNase A (0.1 mg/mL, diluted with PBS, Sigma, USA) for 0.5 h at 37 °C, and 3 additional washes with PBS. The cells were then incubated with 1 mL propidium iodide (PI, 50 μg/mL, diluted in PBS, Sigma, USA) for 0.5 h at 37 °C in a darkened environment, washed 3 times with PBS, and re-suspended in PBS. The cell cycle was analyzed using FACS Calibur (Becton-Dickinson, USA). The resulting data were analyzed using Modfit software^58^.

### Protein expression

Protein expression was analyzed by Western blotting (WB) analysis. Treated cells were harvested with a lysis buffer (P0013, Beyotime, China), and total protein concentrations were analyzed using a BCA Protein Assay Kit (P0011, Beyotime, China). About 30 μg of total protein was separated on a 10% SDS-PAGE gel and transferred onto a polyvinylidene fluoride (PVDF) membrane (FFP39, Beyotime, China) using 200 mA of constant electrical current. The membrane was blocked with TBST buffer (1.21 g of Tris, 5.84 g of NaCl and 1 mL of Tween-20 in 1 L ddH_2_O, pH 7.5) containing 5% skim milk (M/V) for 1.5 h at room temperature (about 25 °C), and then incubated with primary antibody (diluted with TBST containing 5% skim milk) for 1.5 h at room temperature or overnight at 4 °C. The membrane was washed 3 times (5 min/time) with TBST and then incubated with HRP-conjugated secondary antibody (diluted with TBST containing 5% skim milk, 1:2000, Bioss, China) for 1 h at room temperature. The membrane was washed 3 times (10 min/time) with TBST, and the chemiluminescence of HRP was visualized with Super ECL Plus (32106, Thermo, USA). Protein band intensity was analyzed with ImageJ2X software. The β-actin was use as the reference protein. The primary antibodies used were the following: anti-CSN1S1 (1:1000, SAB1401093, Sigma, USA), anti-CSN1S2 (1:500, bs-10034R, Bioss, China), anti-CSN2 (1:500, orb18512, Biorbyt, UK), anti-CSN3 (1:1000, SAB1401094, Sigma, USA), anti-β-actin (1:1000, 4967, Cell Signaling Technology, USA), anti-mTOR (1:1000, 2972, Cell Signaling Technology, USA), anti-p-mTOR (Ser2481) (1:1000, 2974, Cell Signaling Technology, USA), anti-Raptor (1:500, sc-27744, Santa Cruz, USA), anti-S6K1 (1:500, sc-230,Santa Cruz, USA), anti-p-S6K1 (Thr389) (1:500, sc-11759, Santa Cruz, USA), anti-GCN2 (1:500, sc-374609, Santa Cruz, USA) and anti-SESN2 (1:500, sc-292558, Santa Cruz, USA).

### Plasmid construction and transfection

Plasmid construction was performed as previously reported^59^. Total RNA of CMEC was extracted using a Trizol reagent (15596-026, Invitrogen, USA), and the cDNA was synthesized with M-MLV reverse transcriptase (2641 A, Takara, China). The CDS area of SESN2 was amplified with PCR. The SESN2 primers (GenBank: BT021909.1) were the following: Forward: 5′-CGGAATTCCACACCATGATCGTTGCGG-3′, the *EcoR* I site is underlined; and Reverse: 5′-GAAGATCTCAGGTGAGTAAATGGGCTTCC-3′, the *Bgl* II site is underlined. The PCR product was sequenced (BGI, China) and then subcloned into the MCS of eukaryotic expression vector pCMV-C-Flag (D2632, Beyotime, China). The plasmid will be subsequently refered to as SESN2-Flag.

For the SESN2 GO, the transfection of SESN2-Flag was performed. The CMEC were plated into 6 well plates a density of 1.0 × 10^5^ cells per well, and at about 70% confluence, the medium was changed with OPTI-MEM I medium (31985-070, Invitrogen, USA). SESN2-Flag vector and pCMV-C-Flag empty vector were transfected with Lipofectamine 2000 transfection reagent (11668019, Invitrogen, USA) according to the manufacturer’s instructions. Briefly, for cells of each well to be transfected, 5 ìg DNA plasmid and 10 μl Lipofectamine 2000 transfection reagent were diluted into 250 ìl OPTI-MEM I medium, respectively. After incubating for 5 min at room temperature, the diluted DNA plasmid and Lipofectamine 2000 transfection reagent were mixed, and incubated for 20 min at room temperature. Then the mixture was added to well containing cells. After 6 h, the OPTI-MEM I media were switched to DMEM/12 media containing 10% FBS.

### Small interfering RNA transfection

The specific siRNA of genes indicated in this experiment and the negative control siRNA were synthesized (GenePharma, Shanghai, China). The si-SESN2 was transfected using Lipofectamine 2000 transfection reagent according to the manufacturer’s instructions. The operation process was the as same as that of SESN2-Flag DNA plasmid transfection, but the amount of siRNA and Lipofectamine 2000 transfection reagent were 100 pM and 10 μl per well, respectively. The siRNA sequences used in this study are shown in Table 1.

**Table 1:** List of siRNA sequences.

Gene name	Sequences of siRNA (5′-3′)	
SESN2	sense	CCUUUGCAAACCCAGAUAUTT
	antisense	AUAUCUGGGUUUGCAAAGGTT
mTOR	sense	CCACUCGAAUUGGAAGAUUTT
	antisense	AAUCUUCCAAUUCGAGUGGTT
Negative control	sense	UUCUCCGAACGUGUCACGUTT
	antisense	ACGUGACACGUUCGGAGAATT

### Immunofluorescence

The CMEC were plated on cover slips in 6 well plates with the concentration of 1.0 × 10^5^ cells per well and cultured with DMEM/12 containing 10% FBS for 12 h. The cells were treated for 6 h and used for lysosomal localization analysis of mTOR in the following manner. Media were removed. Cells were washed 3 times with PBS, fixed with ice-cold methanol at 4 °C for 10 min, and washed 3 times (5 min/time) with TBST. Cells were blocked with a blocking buffer (P0102, Beyotime, China) for 1 h at 37 °C and then incubated with an anti-mTOR rabbit polyclonal primary antibody [1:200, Cell Signaling Technology, USA, diluted with primary antibody dilution buffer (P0103, Beyotime, China)] and an anti-LAMP2 goat polyclonal primary antibody (1: 100, Santa Cruz, USA, diluted with primary antibody dilution buffer) for 1 h at 37 °C. Cells were subsequently washed 3 times (5 min/time) with TBST and incubated with mouse-anti-rabbit alexa fluor 488-conjugated secondary antibody [Bioss, China, diluted 1:100 with secondary antibody dilution buffer (P0108, Beyotime, China)] and mouse-anti-goat alexa fluor 647-conjugated secondary antibody (Bioss, China, diluted 1: 100 with secondary antibody dilution buffer) for 45 min at 37 °C in the dark. The cells were washed 3 times (5 min/time) with TBST, and incubated with 4′,6-diamidino-2-phenylindole (DAPI) (0.5–10 μg/ml, C1002, Beyotime, China) for 10 min at 37 °C in a dark environment, and then washed 3 times (10 min/time) with TBST. The cells were fixed on the slides with antifade mounting medium (P0126, Beyotime, China) and observed with laser scanning confocal microscopy (LEICA, Germany). Co-localization was analyzed with ImageJ2X software, and at least 10 treated cells in each co-localization were used to analyze the index of co-localization.

### Statistical analysis

Date were analyzed using the Proc Mixed procedure of SAS (version 9.1, SAS Institute, Cary, NC). The fixed effect was treatment for all models. For growth rate measurements which were conducted across 3 cell preparations, cell batch was included as a random effect. For all other measurements, plate was included as a random effect. Least squares means (LSM) of treatments were compared using Tukey’s multiple comparisons procedure, and the effects were considered significant at a probability value of *P* < *0*.*05*. Data were presented as LSM ± standard error of the means (SEM). All LSM represented at least three independent experiments.

## Electronic supplementary material

Supplementary Information
