# Supplementary material for: SESN2 negatively regulates cell proliferation and casein synthesis by inhibition the amino acid-mediated mTORC1 pathway in cow mammary epithelial cells
Source: Sci Rep. 2018 Mar 2;8:3912. doi: 10.1038/s41598-018-22208-w (PMC5834632; doi:10.1038/s41598-018-22208-w)
Supplement: Supplementary file 1 — Supplementary Information [file 41598_2018_22208_MOESM1_ESM.docx]

**SESN2 negatively regulates cell proliferation and casein synthesis by inhibition the amino acid-mediated mTORC1 pathway in cow mammary epithelial cells**

Chaochao Luo^†^, Shengguo Zhao^†^, Muchen Zhang, Yanan Gao, Jiaqi Wang, Mark D Hanigan, Nan Zheng^*^

^†^Chaochao Luo and Shengguo Zhao contributed equally to this paper.

*Corresponding author: Nan Zheng.

**Supplemental Experimental Procedures**

**Isolation of lysosomes and lysosomal localization of mTOR.** The CMEC were cultured in cell culture dishes (10 cm diameter) until 90% confluence. Cells were treated with AA-, EAA+ and AA+ for 10min, 0.5h, 1h, 6h and 12h followed by lysosome isolation using a commercial kit (Sigma, [233-140-8](http://www.sigmaaldrich.com/catalog/search?term=233-140-8&interface=EG/EC%20No.&N=0&mode=partialmax&lang=zh&region=CN&focus=product), USA). The purity of the lysosomes and mTOR in lysosomal extracts was analyzed using WB. The antibodies used in the test were anti-LAMP2 (Lysosome marker, 1:200, sc-8100, Santa Cruz, USA), anti-VDAC1 (Mitochondria marker, 1:200, sc-8829, Santa Cruz, USA ), anti-GM130 (Golgi marker, 1:200, sc-31148, Santa Cruz, USA), anti-calnexin (endoplasmic reticulum marker, 1:200, sc-6465, Santa Cruz, USA), and anti-mTOR (1: 1000, 2972, Cell Signaling Technology, USA).

**Effects of individual AA on mTORC1 signaling and the expression of SESN2.** The CMEC were plated into 6 well plates and grown to 90% confluence. Cells were treated with AA- or AA- plus Leu (Leu+), Ile (Ile+), Val (Val+), Met (Met+), Gln (Gln+), Arg (Arg+), Lys (Lys+), Pro (Pro+), Ala (Ala+) or NEAA+ or AA+ for 6h. The treated cells were harvested with a lysis buffer, and the concentrations of the total proteins were analyzed with a BCA Protein Assay Kit. The expression of p-S6K1, S6K1, GCN2 and SESN2 were analyzed using WB.


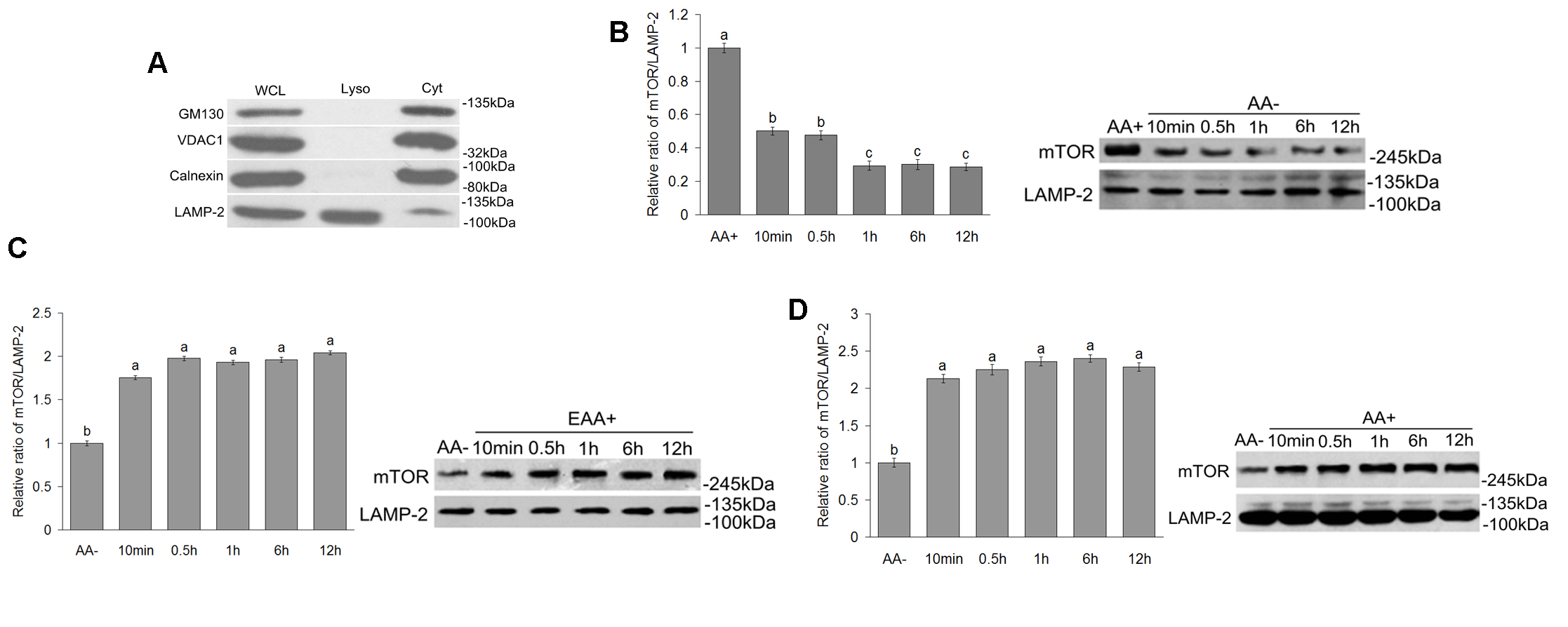


**Supplementary Figure. 1 Localization of mTOR on the lysosomal outer surface in CMEC treated with EAA or AA.**

**(A)** The lysosomes of each group of cells were isolated using a commercial kit (Sigma, USA) and purity of the isolation was tested by western blotting. LAMP2: Lysosome marker, VDAC1: Mitochondria marker, GM130: Golgi marker, Calnexin: endoplasmic reticulum marker, WCL: whole cell lysis, Lyso: lysosome, Cyt: cytoplasmic; **(B)** The localization of mTOR on the lysosomal outer surface of CMEC treated with AA+ and AA- for 10 min, 0.5h, 1 h, 6 h or 12 h was analyzed by WB. The ratio of mTOR/LAMP-2 for the AA+ treatment was used to standardize the data. **(C)** The localization of mTOR on the lysosomal outer surface in CMEC treated with AA- or EAA+ for 10min, 0.5h, 1h, 6h and 12h was analyzed by WB. The ratio of mTOR/LAMP-2 for the AA- treatment was used to standardize the data; **(D)** The localization of mTOR on lysosomal outer surface of CMEC treated with AA- or AA+ for 10 min, 0.5h, 1 h, 6 h or 12 h was analyzed by WB. The ratio of mTOR/LAMP-2 for the AA- treatment was used to standardize the data. Note: LAMP-2 was used as loading control. In the bar charts, different lowercase superscript letters indicate significant differences (*p < 0.05*).


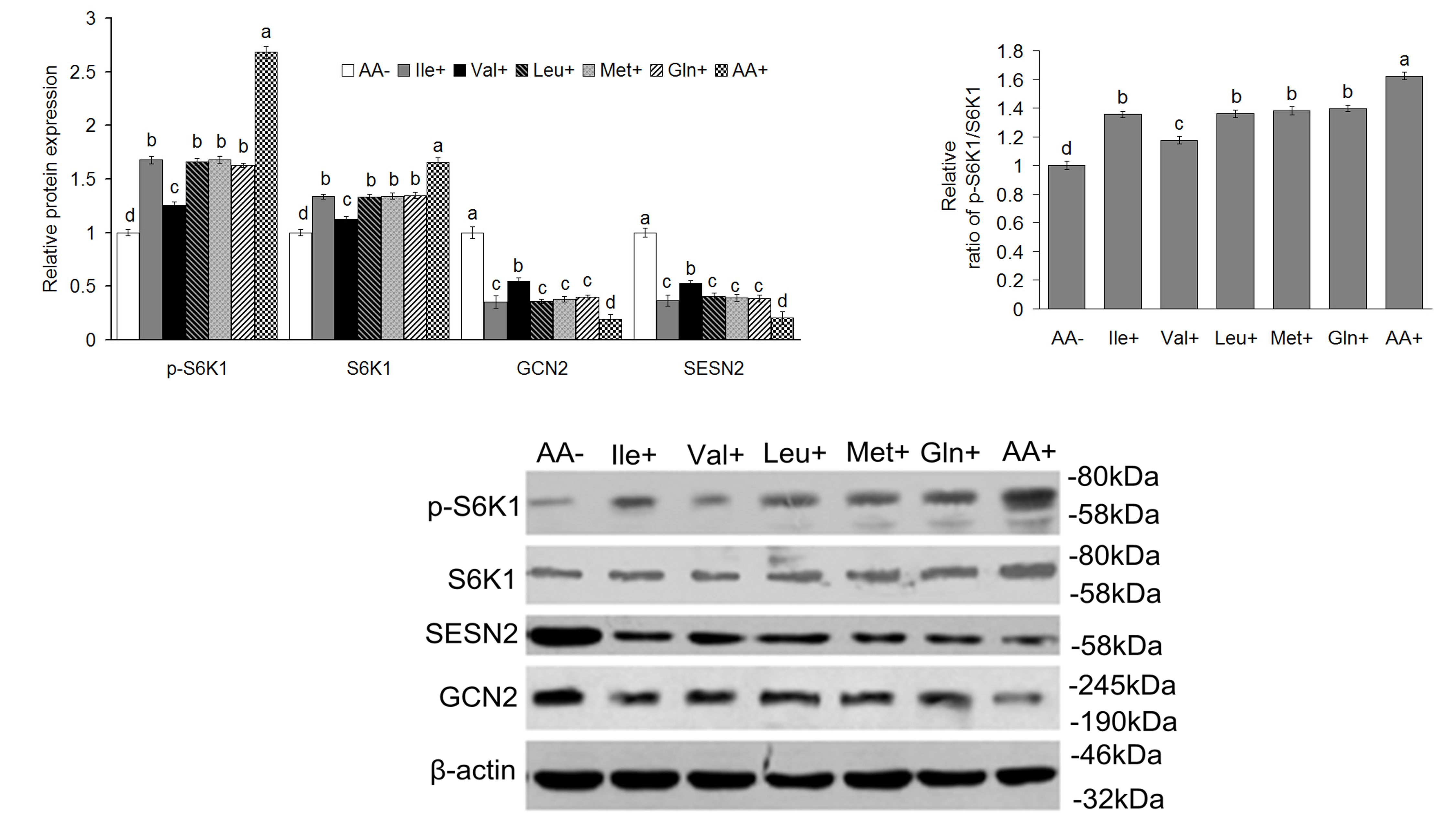


**Supplementary Figure 2 The regulation of SESN2 and mTORC1 signaling by individual AA.** Expression of p-S6K1, S6K1, SESN2 and GCN2, and the phosphorylation ratio of S6K1 of CMEC treated with AA- or AA- plus Ile+, Val+, Leu+, Met+, Gln+ or AA+ was analyzed by WB. The ratio of p-S6K1/S6K1 for the AA- treatment was used to standardize the data. Note: In the bar charts, the different lowercase superscript letters indicate significant differences (*p < 0.05*).


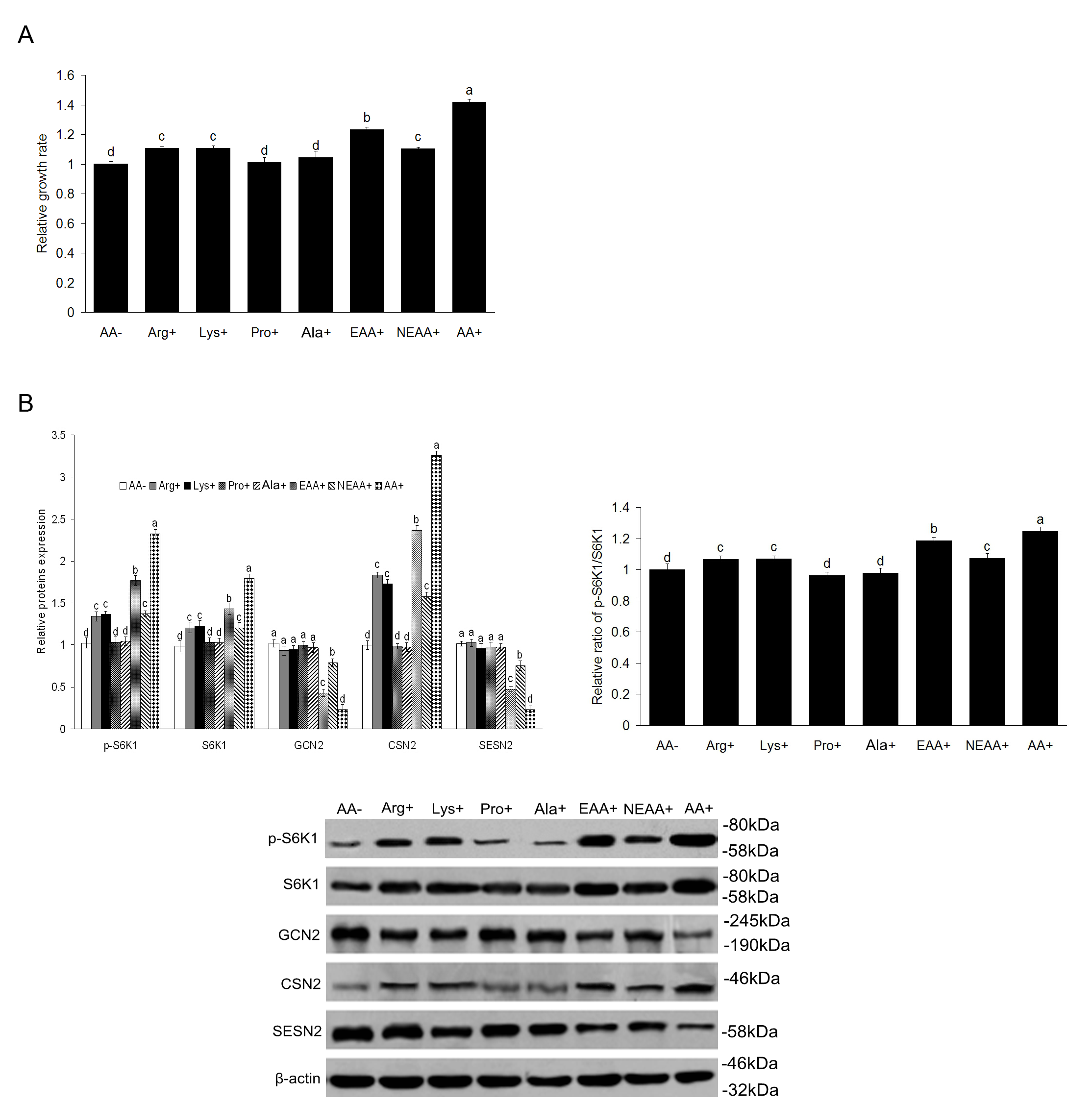


**Supplementary Figure 3 The regulation of cell growth, SESN2 expression and mTORC1 signaling by EAA, NEAA and individual AA. (A)** Cell growth of CMEC treated with AA- or AA- plus Arg+, Lys+, Pro+, Ala+ or EAA+ or NEAA+ or AA+ was analyzed by MTT assay, the data of “AA- group” was defined as “1”; **(B)** Expression of p-S6K1, S6K1, GCN2, CSN2 and SESN2, and the phosphorylation ratio of S6K1 of CMEC treated with AA- or AA- plus Arg+, Lys+, Pro+, Ala+ or EAA+ or NEAA+ or AA+ was analyzed by WB. The ratio of p-S6K1/S6K1 for the AA- treatment was used to standardize the data. Note: In the bar charts, the different lowercase superscript letters indicate significant differences (*p < 0.05*).


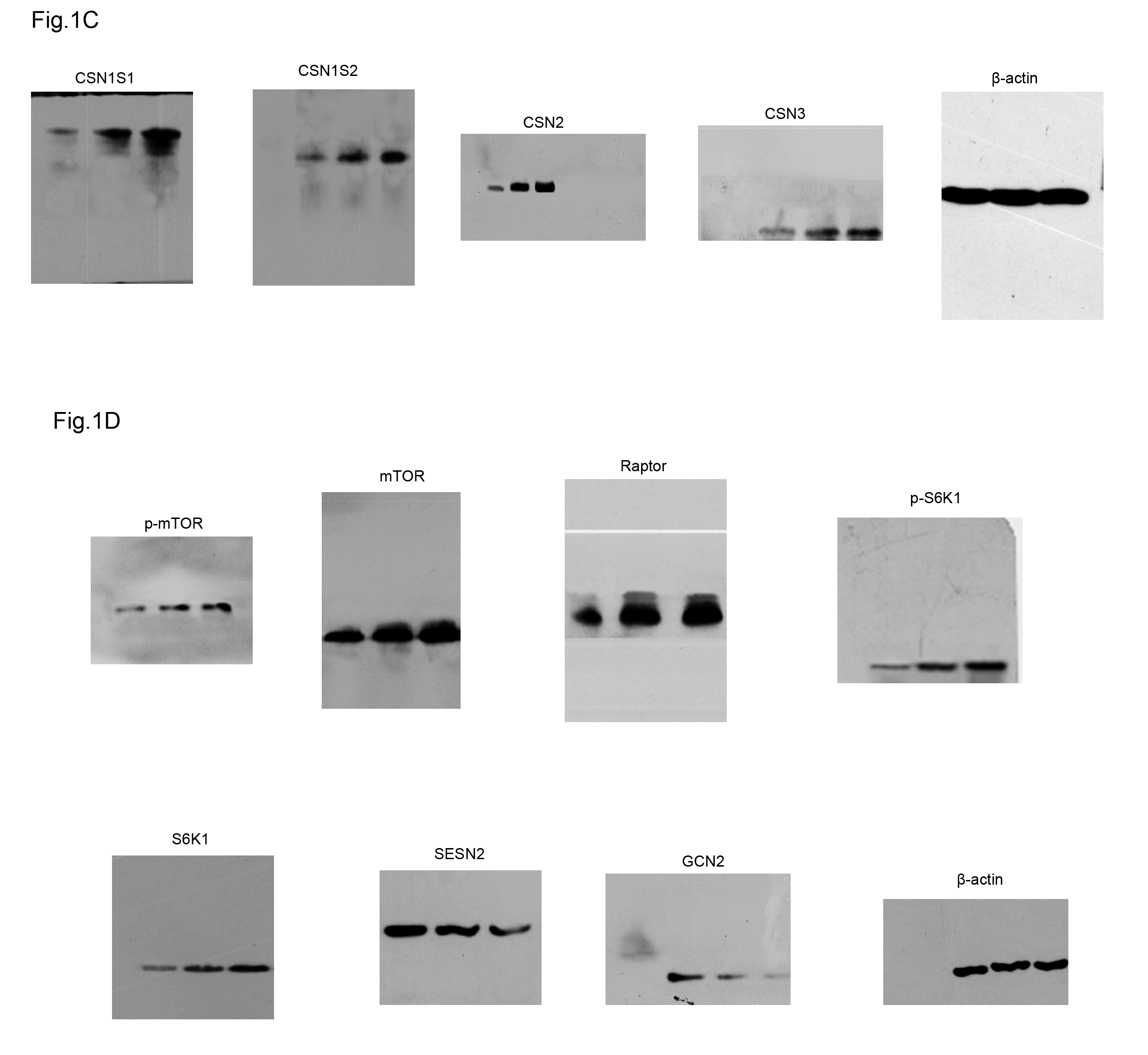

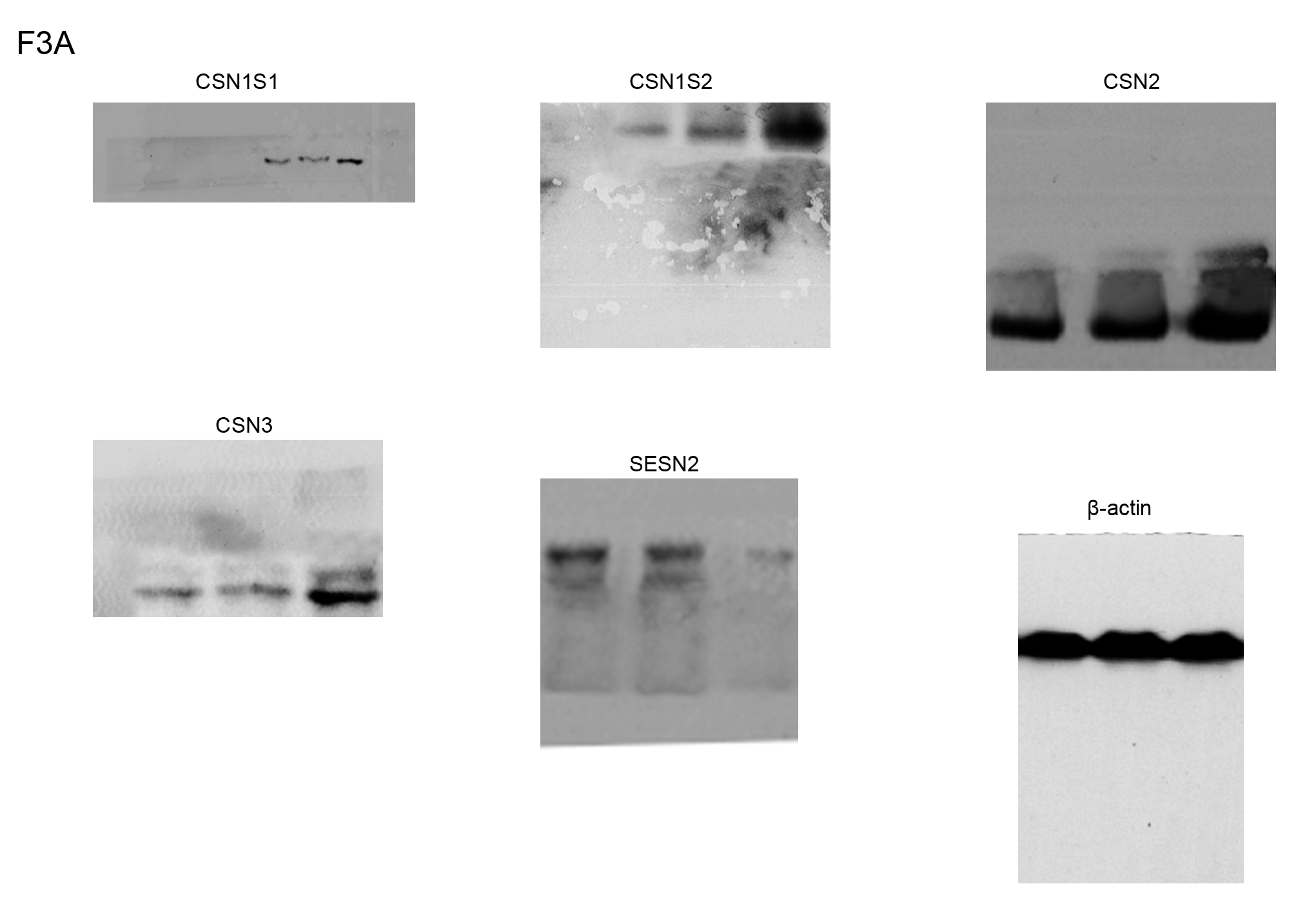

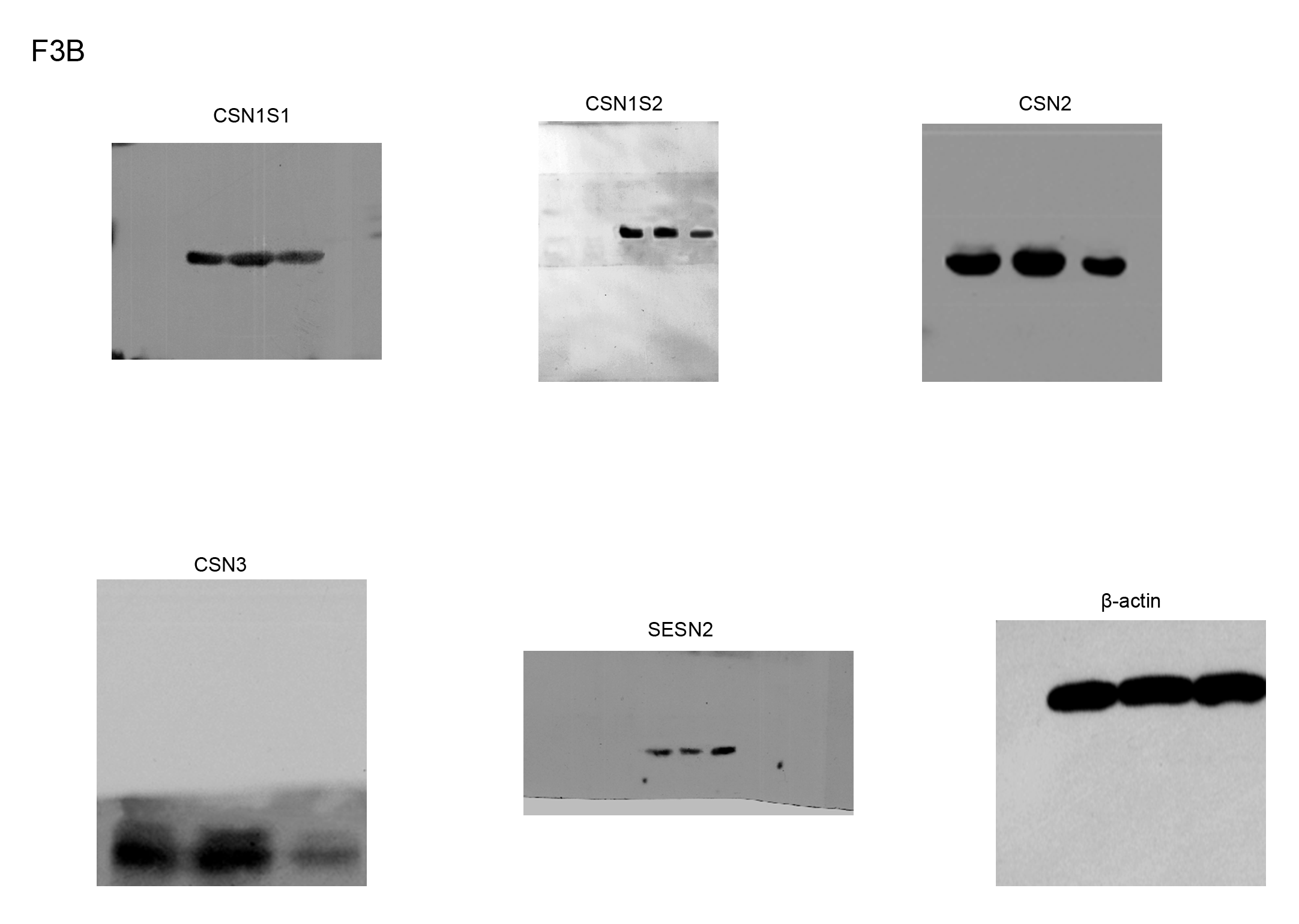

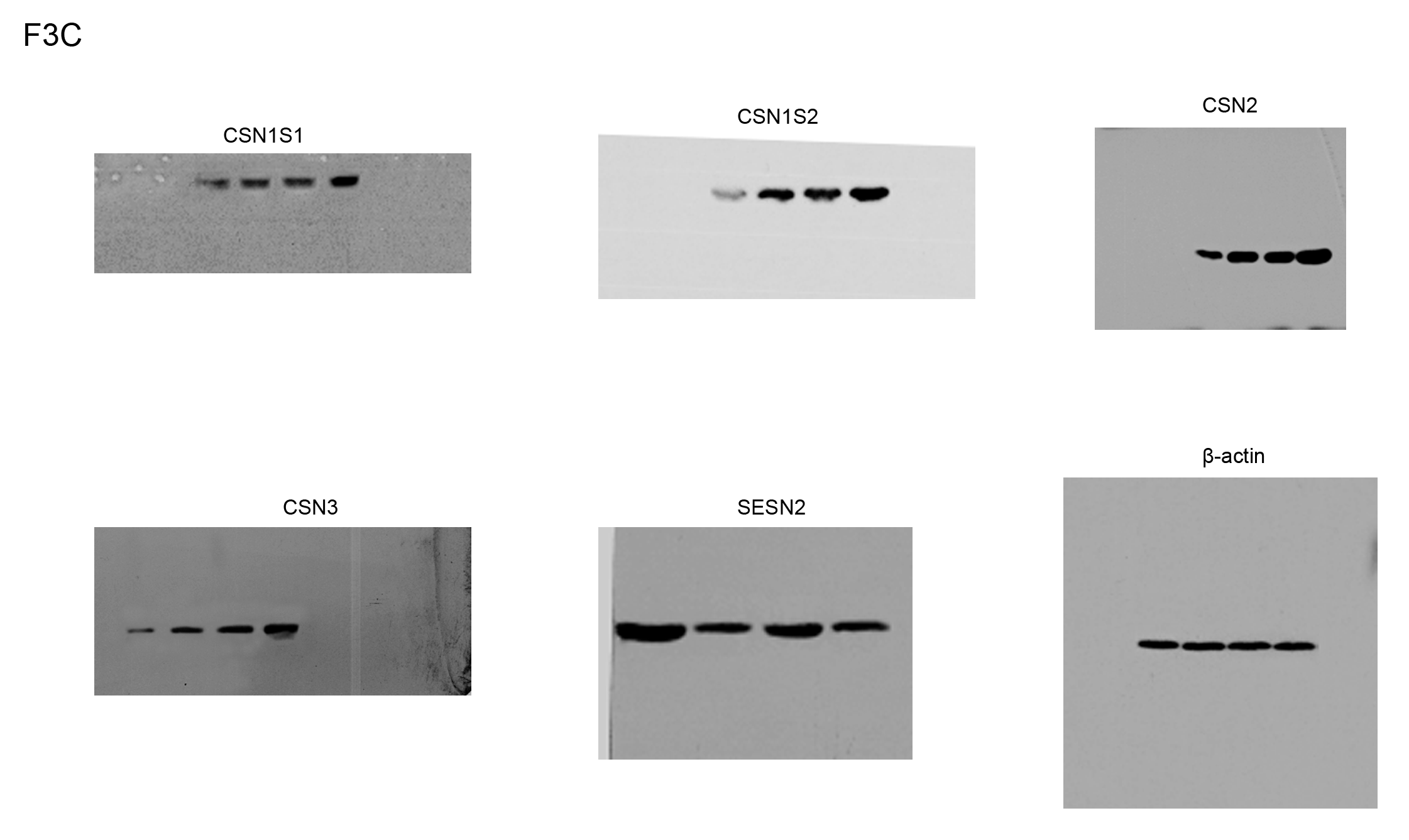

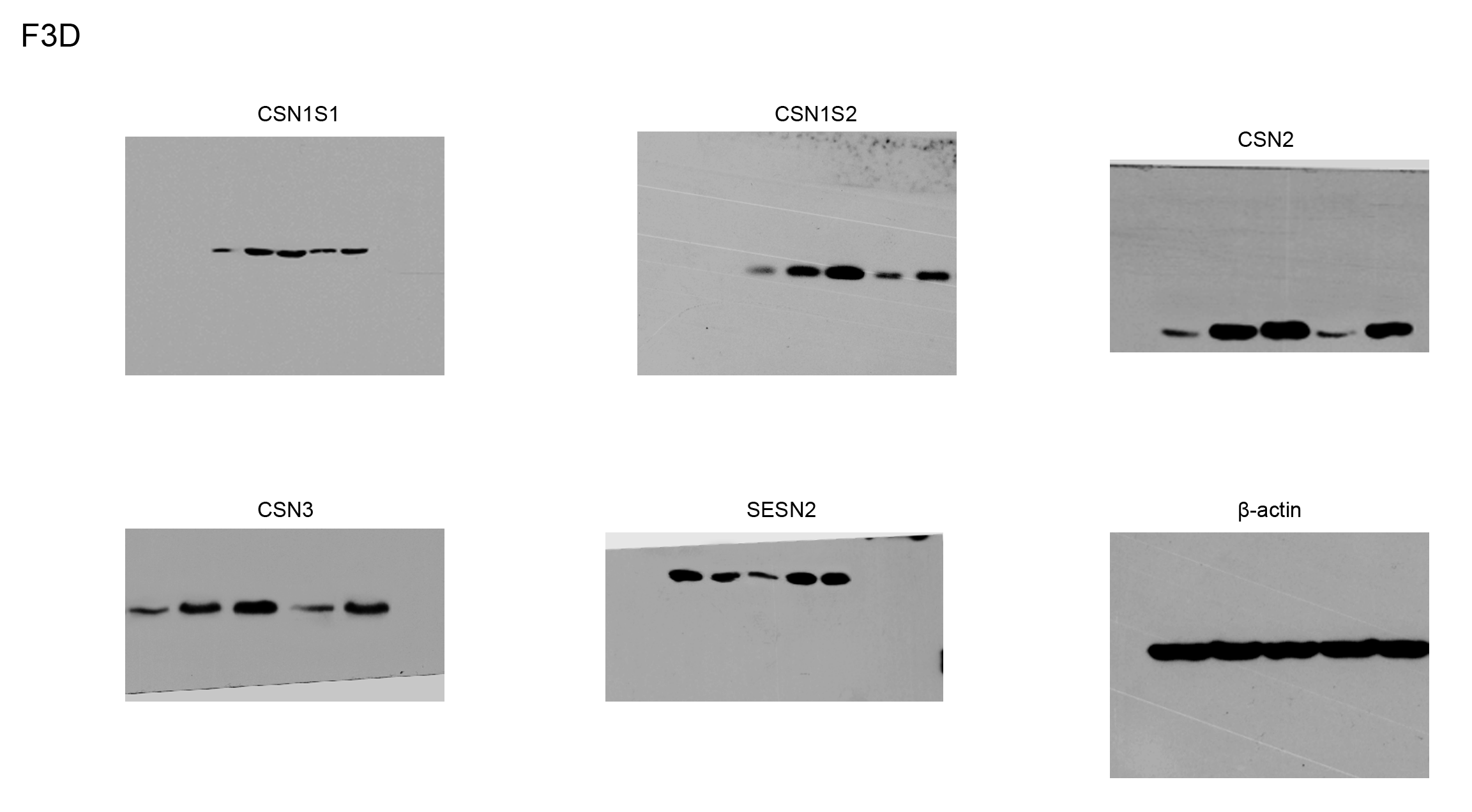

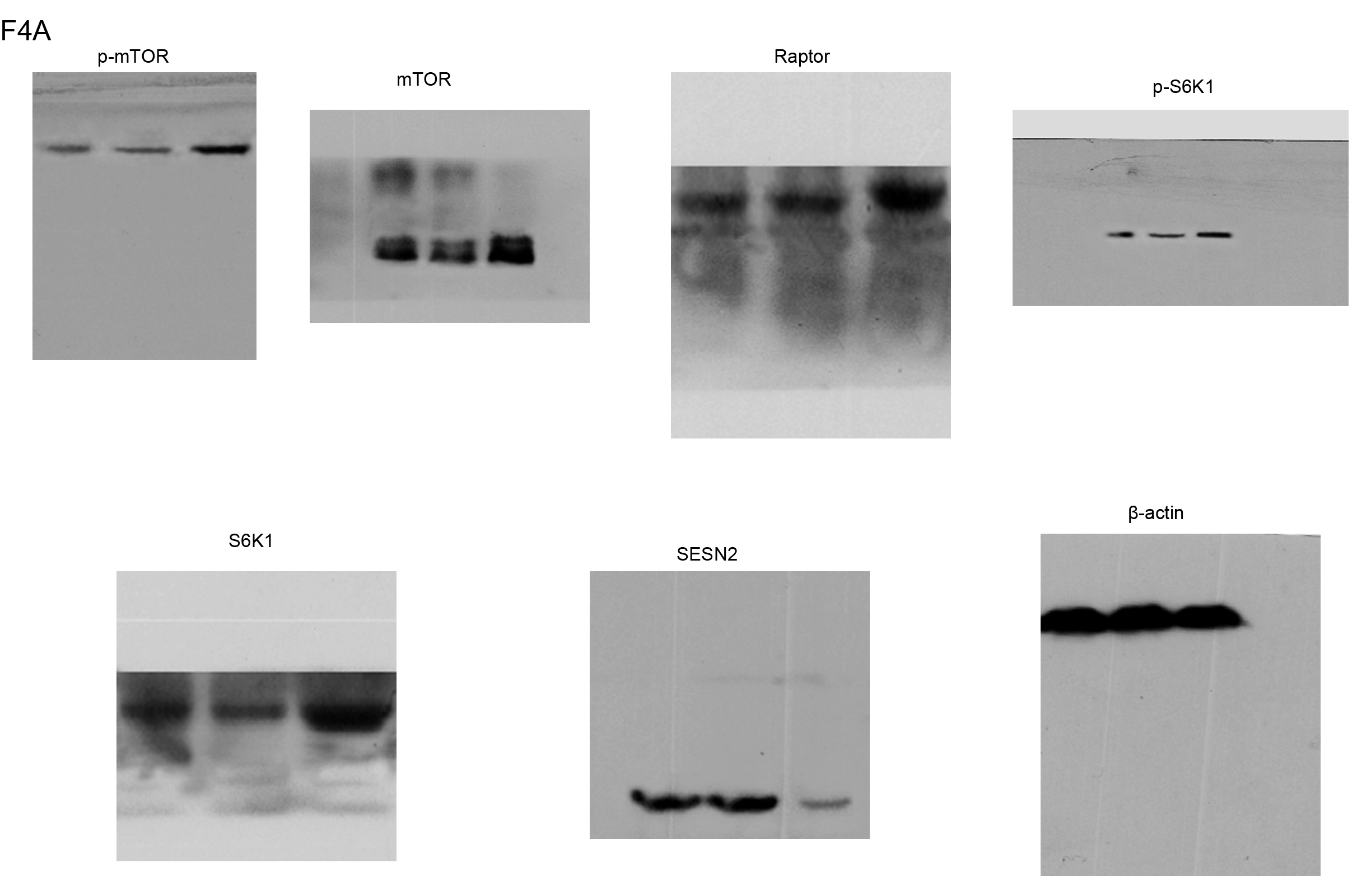

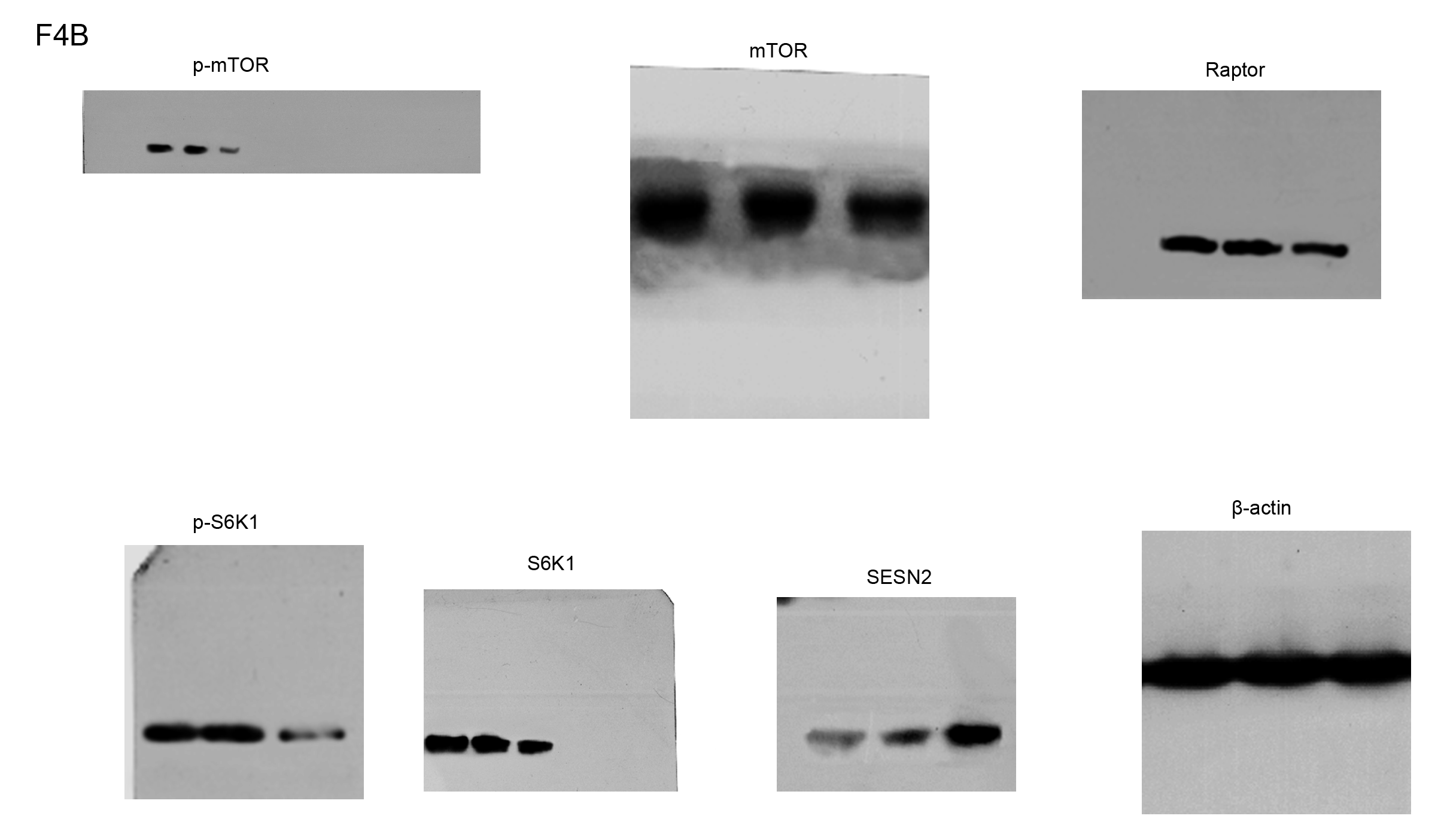

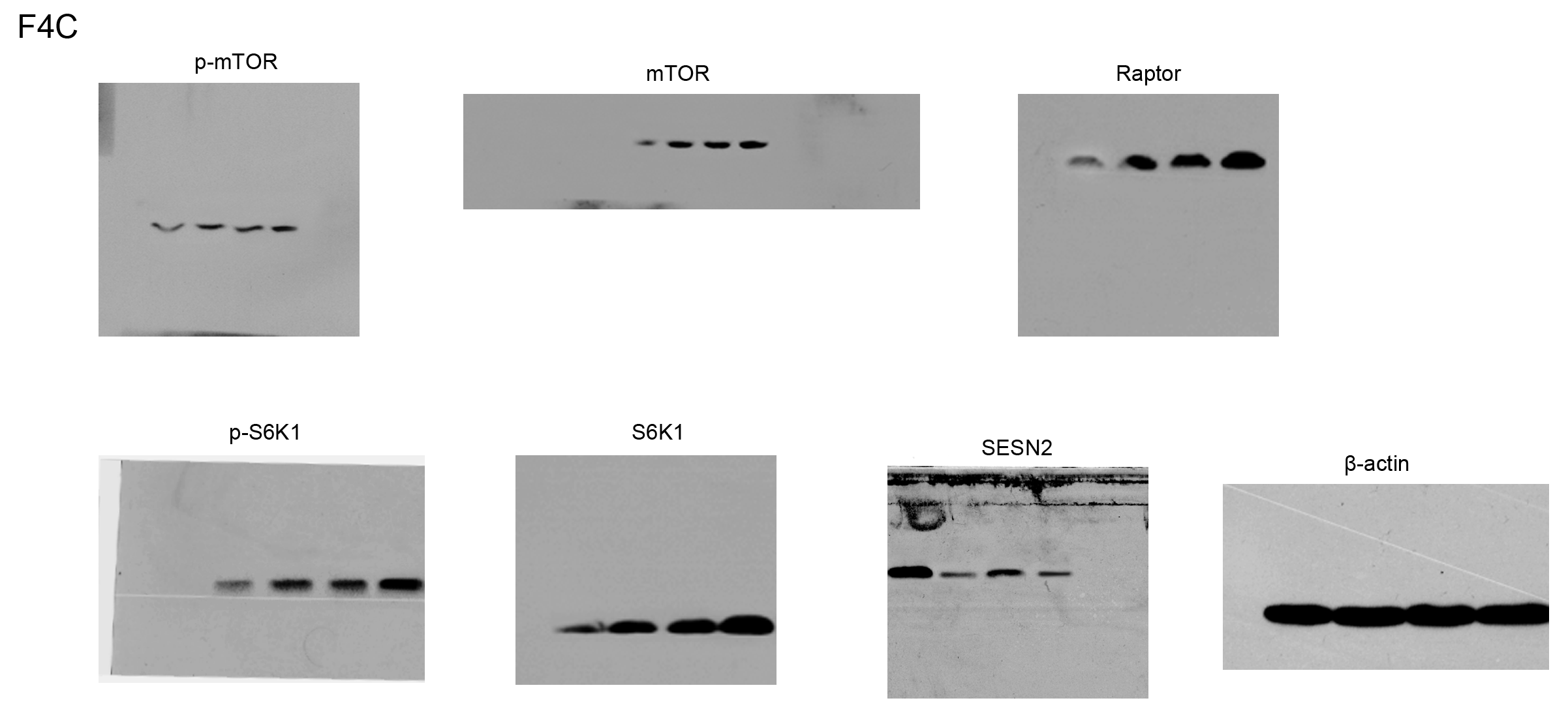

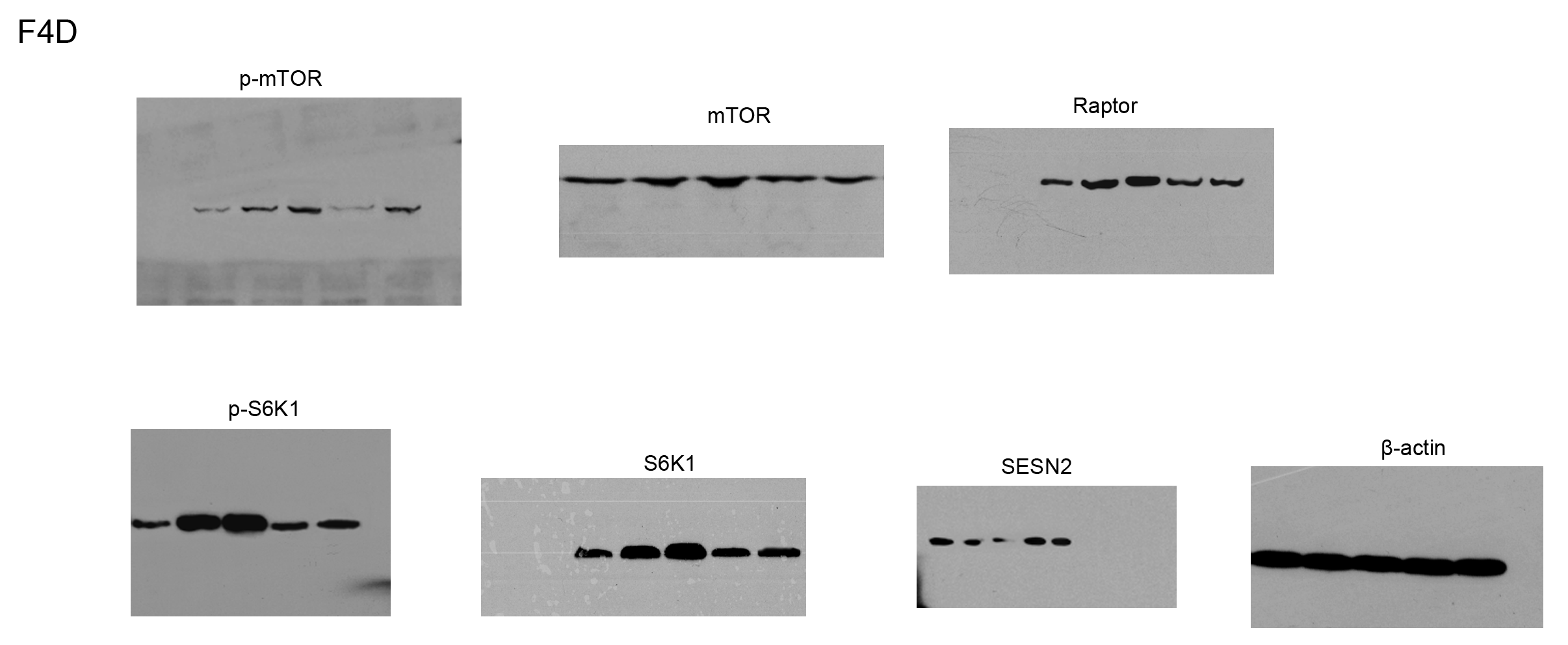

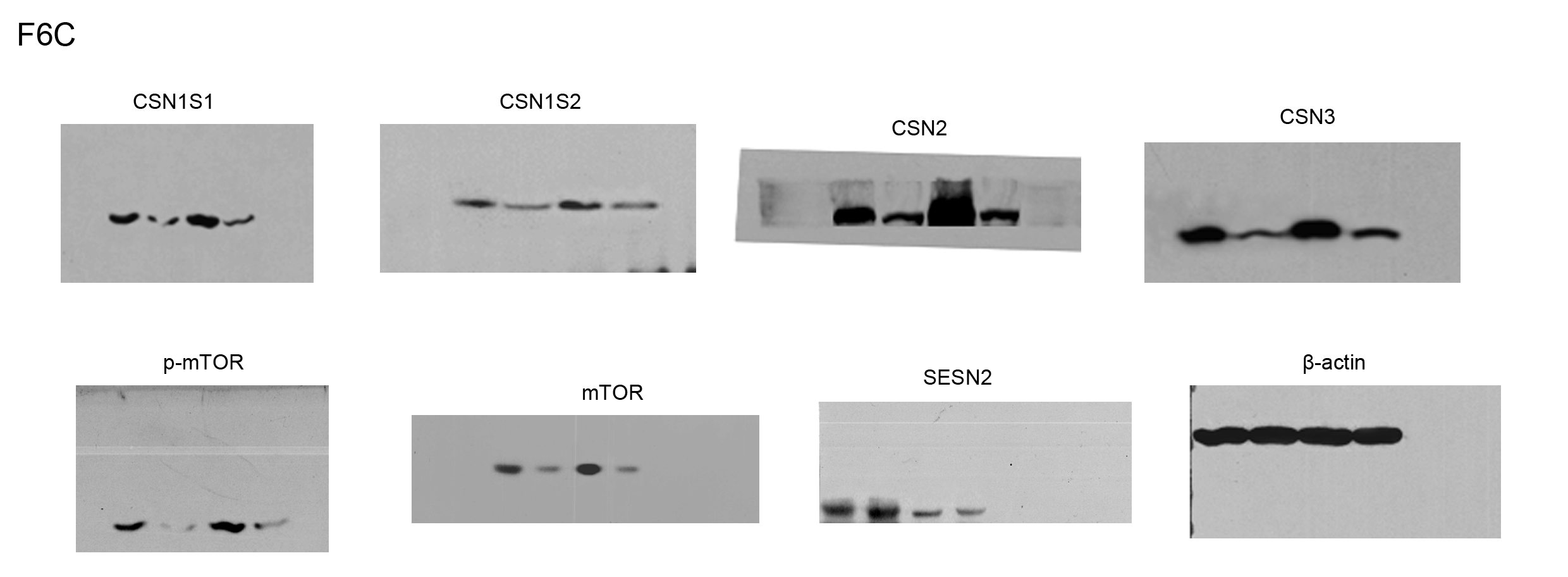


**Supplementary Figure 4.** Uncropped images for all Western blots displayed in main figures.
